# Toward Sustainable Lunar Construction: Optimization of Composite Alkali Activators for Nano-SiO_2_-Modified Simulated Lunar Soil-Based Geopolymer with Macro- and Microstructural Characterization

**DOI:** 10.3390/ma19183882

**Published:** 2026-09-11

**Authors:** Zhenyu Wang, Liqing Li, Faping Li

**Affiliations:** 1 School of Civil Engineering and Architecture, Henan University of Technology, Zhengzhou 450001, China; wzy92642000@163.com (Z.W.); llq@haut.edu.cn (L.L.); 2 School of Civil Engineering, Wuhan University, Wuhan 430072, China

**Keywords:** simulated lunar soil-based geopolymer, nano-silica, sustainability, mechanical properties, microstructure

## Abstract

**Highlights:**

- Nano-SiO_2_ improves mechanical properties at reduced alkali-activator dosages.
- NaOH may promote precursor depolymerization.
- Ca(OH)_2_ may facilitate polycondensation.
- Activator ratios influence gel composition, pore structure, and mechanical performance.

**Abstract:**

Lunar soil-based geopolymers are promising in situ construction materials, but their reliance on Earth-transported alkali activators limits sustainability. This study developed a nano-SiO_2_-modified simulated lunar soil-based geopolymer (NS-SLSG) with reduced activator demand by optimizing sodium hydroxide (SH), calcium hydroxide (CH), and sodium silicate (SS). Inductively coupled plasma atomic emission spectrometry (ICP-AES), orthogonal testing, Fourier transform infrared spectroscopy (FT-IR), X-ray diffraction (XRD), Scanning electron microscopy (SEM), Thermogravimetry–differential scanning calorimetry–derivative thermogravimetry (TG-DSC-DTG), Energy-dispersive X-ray spectroscopy (EDS), and Mercury intrusion porosimetry (MIP) were used to evaluate apparent elemental-release characteristics, flowability, mechanical properties, and microstructure. The experimental results indicated that the measured Al and Si concentrations were particularly sensitive to the SH dosage; SH and SS exerted comparatively greater effects on flowability, whereas SH and CH had greater effects on mechanical performance. Adding 0.75 wt.% NS reduced the total admixture mass in the experimental formulation by 23.9% while increasing compressive and flexural strengths by 26.0% and 19.5% to 36.15 and 8.26 MPa, respectively. Microstructural results suggested that SH promoted depolymerization, while CH may have enhanced Ca^2+^-assisted polycondensation, with corresponding variations in gel composition and pore structure. These findings indicate a viable strategy for improving the resource efficiency of lunar construction materials.

## 1. Introduction

The Moon, as a natural satellite of the Earth, holds crucial information about the early evolution of the solar system. The lunar soil is rich in helium-3 (fusion fuel), rare earth elements, and other valuable resources. Establishing a lunar research station can provide a stable platform for astronomical observation, space science experiments, and resource collection. To this end, National Aeronautics and Space Administration (NASA) plans to deploy long-term satellites in lunar orbit via the Earth–Moon Lagrangian points to explore the outer layer of the Moon, surface, and underground environment [1]. The European Space Agency (ESA) has announced plans for a “Lunar Village” [2], while Russia aims for deep exploration of the south polar region of the Moon through the Luna25–29 series of missions [3]. The China National Space Administration (CNSA) intends to establish a basic lunar research station model by 2028 [4]. As lunar exploration advances, in situ construction technology [5] (ISRU) has become essential for creating a sustainable lunar research station, significantly reducing transportation costs between the Earth and the Moon.

To effectively address the technical challenges of in situ construction of building materials on the lunar surface, many researchers have developed two types of in situ curing materials based on the mineral composition and chemical reactivity of lunar soil: thermally cured [6,7] and chemically cured types. Thermal curing relies on high temperatures to melt low-melting-point phases such as glassy components in lunar soil, forming a liquid phase that bonds high-melting-point crystalline phases (e.g., olivine and pyroxene) and achieves densification through cooling and crystallization. This approach offers excellent mechanical properties and high in situ resource utilization, but the melting process consumes substantial energy, which may exacerbate the power supply pressure during the early stages of lunar research station construction and thus limit its applicability for lunar-surface construction to some extent. Chemical curing is typically represented by lunar soil-based geopolymers, in which alkali activators dissociate to release high concentrations of OH^−^ ions that attack the aluminosilicates in lunar soil, releasing reactive monomers, which subsequently undergo polycondensation to form a covalently bonded Si-O-Si/Al network. This chemical curing process is regulated by multiple parameters, including the type, concentration, modulus, liquid-to-solid ratio, and curing temperature of the alkali activator, enabling the design and optimization of the chemical composition and microstructure of the resulting gel. Owing to their low energy consumption, high durability, rapid strength development, ease of construction, and excellent compatibility with the composition of lunar soil, lunar soil-based geopolymers have gradually been regarded as one of the ideal candidate materials for lunar-surface building structures [8,9,10,11,12,13].

Wu et al. [14] explored the activation of simulated lunar soils (LRS1-LRS3) using sodium hydroxide (SH) and sodium silicate (SS). Their findings indicated that SH effectively activated the glass-rich simulated lunar soil (LRS1), achieving a compressive strength of 28.3 MPa, while SS was better for the low glassy phase (LRS2) with a compressive strength of 23.9 MPa. This suggests that the choice of alkali activator depends on the composition of the soil. Li et al. [15] demonstrated that solid SS as a composite alkali activator significantly influences the mechanical properties of simulated lunar soil-based geopolymer (SLSG) at 60 °C, identifying an optimal formulation of 18 wt.% of SS and 0.25 hydrogel, achieving a 12-day compressive strength of 30.54 MPa. Zhang et al. [16] used SH as an alkali activator to prepare SLSG and assessed its mechanical properties under simulated lunar-surface temperature cycling. Results showed that high temperature enhances SLSG modeling, achieving flexural and compressive strengths of 5.7 and 31.2 MPa, respectively. Notably, the precursor type (chemical composition, physical phase composition) and alkali activator (type, concentration, and doping ratio) significantly influence the mechanical properties of SLSG. However, the effects of various alkali activators, such as calcium hydroxide (CH) and composite activators, on lunar soil material activation, mechanical properties, and the mechanism at play require further investigation.

Although SLSG exhibits good mechanical properties and thermal cycling stability [17], the reliance on alkali activators limits in situ resource utilization compared to molding techniques such as high-temperature sintering [18] and lunar soil bags [19]. Researchers have extensively analyzed the effects of various admixtures on the mechanical properties and mass–strength efficiency of SLSG. Zhou et al. [20] found that adding 5 wt.% and 10 wt.% of Al_2_O_3_ increased 28-day compressive strength of SLSG by 45.9% and 46.0%, respectively, though the higher dosage reduced mass–strength efficiency. Zhang et al. [21] observed that 0.3 wt.% carbon nanofiber (CNF) improved flexural strength of SLSG by 34.8% and toughness by 83.9%. Shao et al. [22] demonstrated that 0.6 wt.% hybrid steel-polypropylene fibers improved compressive strength by 22.0% and mass–strength efficiency by 19.9%. Wang et al. [23] showed that 0.75 wt.% nano-silica (NS) increased compressive strength by 40.8%, flexural strength by 57.7%, and the respective efficiencies by 36.9% and 54.8%, respectively. These studies confirm the effectiveness of admixtures in enhancing mechanical properties and mass–strength efficiency of the SLSG but do not address their potential in reducing alkali-activator dosage or the relationships between dosage and enhancement. The study by Hu [24] demonstrated that NS exhibits better dispersion stability and enhanced performance in alkaline environments, which aligns well with the high alkalinity required for activating simulated lunar soil. Given that NS is a highly reactive nanomaterial capable of enhancing the reactivity of simulated lunar soil and promoting geopolymer gel phase formation [25], it shows promise in compensating for the reduced activation efficiency at lower alkali-activator dosages, thereby enabling a reduction in the total admixture mass. Considering the high cost of Earth–Moon transportation [8], even a 1 wt.% reduction in the total admixture—equivalent to a mass saving of approximately 10 kg per ton of manufactured building material—would yield significant economic benefits.

Collectively, existing studies have demonstrated that both alkali-activator composition and reinforcing additives can effectively regulate the reaction and mechanical performance of SLSG; however, the potential of NS to compensate for the insufficient activation associated with reduced composite activator dosages, as well as the underlying relationships linking the apparent elemental-release characteristics under different alkali-activation conditions and slurry flowability to gel evolution, pore-structure development, and macroscopic properties, remains insufficiently understood. On this basis, this study aims to optimize the composite alkali-activator ratio of SH, CH, and SS in order to modulate the chemical reaction pathways and to harness the reactive enhancement mechanism of NS, thereby improving the mechanical properties of NS-SLSG while reducing the activator dosage. The apparent release characteristics of Si, Al, and Ca under activation by SH, CH, and SS were analyzed via inductively coupled plasma atomic emission spectrometry (ICP-AES). A three-factor, three-level orthogonal experimental design L9(33) was employed to examine the effects of these components on the flowability and mechanical properties of NS-SLSG. Comprehensive analysis using spectroscopy (FT-IR), X-ray diffraction (XRD), scanning electron microscopy (SEM), thermogravimetric-differential scanning calorimetry-derivative thermogravimetry (TG-DSC-DTG), energy-dispersive X-ray spectroscopy (EDS), and mercury intrusion porosimetry (MIP) analyses elucidated the regulatory mechanisms of composite alkali-activator components on the mechanical properties and microstructure of NS-SLSG. This study may provide a useful reference for the development of high-strength lunar construction materials with reduced activator dosage, and could offer potential cost benefits and improved resource sustainability for deep-space construction applications.

## 2. Materials and Methods

### 2.1. Raw Materials

In this experiment, Tongji-1 (TJ-1) simulated lunar soil [26], which has been widely used in Chang’e series and manned lunar landing projects, was selected as the precursor material. Its parent material originated from the Cenozoic volcanic clastic rock layer in Jingyu County, Baishan, Jilin Province, China, and its mineral structure and chemical composition are similar to Apollo 14 and Chang’e-5 (CE-5) lunar soil samples [27,28].

**Figure 1:**
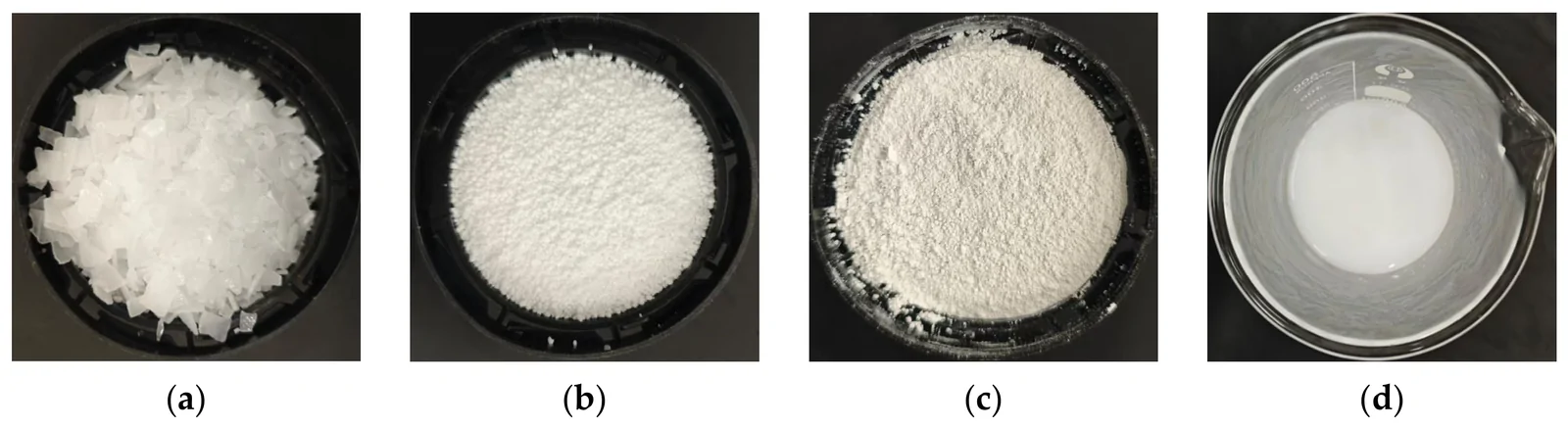
Raw materials. (**a**) SH; (**b**) SS; (**c**) CH; (**d**) NS.

The stringent constraints on both volume and mass for Earth–Moon transportation necessitate the careful selection of material forms. The use of aqueous forms of SH and SS would incur significant penalties in both volumetric efficiency and transportation cost, in addition to posing potential stability challenges during long-distance transit. Therefore, all alkali activators employed in this study were in solid form to enhance transport feasibility and operational reliability. Photographs of the raw materials are provided in Figure 1. The composite alkali-activator system consisted of flake SH (NaOH, 99% purity) from Inner Mongolia Junzheng Chemical and Energy Group Co., Ltd., Wuhai, China; SS (Na_2_SiO_3_·9H_2_O, 95% purity) with a Na_2_O content of 19.3–22.8% and a modulus of 1.03 ± 0.03, from Tianjin Huasheng Chemical Reagent Co., Ltd., Tianjin, China; and CH (Ca(OH)_2_, 95% purity). The NS used in this study was a VK-Sp30W aqueous dispersion with an average particle size of 30 nm and a solid content of 15 wt.% from Zhejiang Zhitai Nanomicro New Material Co., Hangzhou, China. Pure water was used for the tests.

### 2.2. Experimental Design

The apparent release characteristics of Si, Al, and Ca under unitary and ternary activation by SH, CH, and SS were compared using a one-way test. The alkali-activator dosages for the ICP-AES test groups are listed in Table 1.

A three-factor, three-level orthogonal experimental design, designated as L9(33), was implemented with SH, CH, and SS as the variables to analyze their effects on the flowability and mechanical properties of NS-SLSG. The dosage of NS and the initial levels of the three alkali activators were determined based on previous research findings [23]. The NS dosage was fixed at 0.75 wt.% for all NS-containing mixtures and was calculated based on the dry mass of NS relative to the dry mass of TJ-1 simulated lunar soil, rather than the total mass of the commercial NS dispersion. The total water-to-binder ratio was maintained at 0.27 for all mixtures. The total water content included both the water contained in the NS aqueous dispersion and the water used to prepare the composite alkali-activator solution. Accordingly, the amount of water added during the preparation of the composite alkali-activator solution was adjusted by considering the water introduced by the NS dispersion, thereby maintaining the specified total water-to-binder ratio.

To investigate the ability of NS to optimize the resource utilization of SLSG, a baseline formulation with these initial levels was established. The dosage of alkali activators was then progressively reduced from this baseline to thoroughly analyze the potential of NS to compensate for reactivity at lower alkali-activator contents. The specific experimental formulations are presented in Table 2. As shown in Table 2, the composite alkali-activator dosage was categorized into three gradient levels. The initial levels of SH, CH, and SS correspond to the high dosage level, specifically 10 wt.%, 6 wt.%, and 6 wt.%, respectively. The medium dosage level was set at 8 wt.% for SH, 4 wt.% for CH, and 4 wt.% for SS, while the low dosage level was further reduced to 6 wt.% for SH, 2 wt.% for CH, and 2 wt.% for SS. P0 represents the reference group P10-6-6 without NS, and P1 represents the reference group P8-6-2 without NS.

### 2.3. Specimen Preparation

Combined with the established results [23], the preparation process of NS-SLSG was as follows:

(1) The composite alkali activator was prepared by mixing SH, CH, and SS with water at the specified proportions and stirring the mixture for 20 min.
(2) The simulated lunar soil was poured into the cementitious sand mixer (JJ-5, Tianjin Huatong Experimental Instrument Factory, Tianjin, China) at low speed (140 ± 5 r/min) for 1 min.
(3) The NS dispersion and composite alkali activator were slowly poured into the mixer at the same time while mixing at low speed for 4 min.
(4) The geopolymer slurry was poured into three-gang prism molds (160 mm × 40 mm × 40 mm; Cangzhou Shengshi Huaze Instrument Co., Ltd., Cangzhou, China) and vibrated on a vibrating table (NLD-3, Nanjing Jintong Civil Engineering Instrument Research Institute, Nanjing, China) until no visible air bubbles remained. The specimens were initially cured in the molds in an oven (Shaoxing Shangcheng Instrument Manufacturing Co., Ltd., Shaoxing, China) at 100 °C for 24 h. They were subsequently demolded and returned to the same oven, where they were continuously cured at 100 °C until the designated testing ages of 7 and 28 days. During the post-demolding curing period, the specimens were stored without additional sealing and exposed to the oven atmosphere.

### 2.4. Flowability Test

The slurry flowability test was performed in accordance with “Method for Determination of Cementitious Sand Flowability” (GB/T2419-2005) [29]. The process involves placing freshly prepared geopolymer slurry on a standard vibration table (NLD-3, Nanjing Jintong Civil Engineering Instrument Research Institute, Nanjing, China) at 60 vibrations/min for 25 cycles. After stabilization, measure the maximum diameter of its extension in the orthogonal direction, and calculate the average value as the flowability index.

### 2.5. Mechanical Property Testing

The mechanical properties of NS-SLSG were tested per GB/T 17671-2021 “Test Method for Strength of Cement Mortar” [30] using a flexural-compression testing machine (DYE-600S-20, Wuxi Jiangong Testing Instrument Co., Ltd., Wuxi, China). Flexural strength was determined via the three-point bending method. Specimens measuring 160 mm × 40 mm × 40 mm were tested with a span of 100 mm at a loading rate of 50 N/s. For each mixture proportion, three parallel specimens were tested. The specimens from the flexural tests were then subjected to compressive strength testing. These specimens were centered in the compression test fixture and loaded at a rate of 2.4 kN/s, with six specimens tested per group. For both flexural and compressive strength results, any individual measurement deviating by more than 15% from the group average was considered an outlier and excluded from the final calculation. The mean value of the remaining measurements was reported as the final strength result for each group, and the standard deviation of these values was used to represent the error bars in the corresponding figures.

### 2.6. Microstructural Characterization

ICP-AES (Agilent 5110, Agilent Technologies, Santa Clara, CA, USA) was used to measure the Si, Al, and Ca concentrations obtained under different composite alkali-activator conditions. A mixture of 10 g of soil and alkali-activator solution was electromagnetically stirred for 30 min, rested for 3-day, clarified via centrifugation, and the supernatant was tested.

FT-IR spectra were recorded using a Shimadzu IRTracer-100 spectrometer (Shimadzu Corporation, Kyoto, Japan). The samples were prepared using the KBr pellet method, and the spectra were collected over the wavenumber range of 4000–400 cm^−1^ at a spectral resolution of 4 cm^−1^.

The crystalline phase composition was characterized by X-ray diffraction (XRD) using a Bruker D8 Advance diffractometer (Bruker AXS, Karlsruhe, Germany) equipped with Cu Kα radiation (λ = 0.15406 nm) and operated at 40 kV and 40 mA. The diffraction patterns were recorded over a 2θ range of 5–90° at a scan rate of 2°/min. The crystalline phases were identified by matching the experimental diffraction patterns with standard reference patterns in the International Centre for Diffraction Data Powder Diffraction File (ICDD PDF) database.

TG-DSC-DTG (NETZSCH STA 449 F5, NETZSCH-Gerätebau GmbH, Selb, Germany) was used to characterize the thermal mass-loss behavior and thermal stability of NS-SLSG from 50 to 1000 °C at a heating rate of 10 °C/min under an N_2_ atmosphere.

SEM–EDS observations were conducted on the fracture surfaces of the specimens generated after mechanical testing. Prior to SEM–EDS analysis, the specimens were sputter-coated with a thin layer of gold (Au) to improve their electrical conductivity. SEM images were acquired using a ZEISS Sigma 300 (Carl Zeiss Microscopy GmbH, Jena, Germany) scanning electron microscope, and EDS analysis was performed using an Oxford Instruments ULTIM MAX 40 detector (Oxford Instruments NanoAnalysis, High Wycombe, UK). The accelerating voltage was 10.0 kV, and the system and electron-gun vacuum pressures were approximately 4 × 10^−5^ and 2 × 10^−9^ mbar, respectively. Owing to differences in the surface topography of the specimens and observation areas, the working distance was slightly adjusted within the range of 5.9–8.2 mm to optimize focusing and image quality. All SEM images presented in this study were acquired in secondary-electron imaging mode. Backscattered-electron (BSE) images and their corresponding EDS elemental maps were not acquired. The EDS data reported herein were obtained through point analyses of representative dense regions on the specimen fracture surfaces, with at least 10 points analyzed for each mixture.

MIP (Micromeritics AutoPore IV 9520, Micromeritics Instrument Corporation, Norcross, GA, USA) analyzed pore structure with mercury intrusion pressure (0.5–33,000 PSI).

## 3. Results and Discussion

### 3.1. ICP-AES Analysis

The reactivity of simulated lunar soil is related to the availability of Si-, Al-, and Ca-containing species [31], as alkaline environments can influence the release and subsequent reaction of these species.

**Figure 2:**
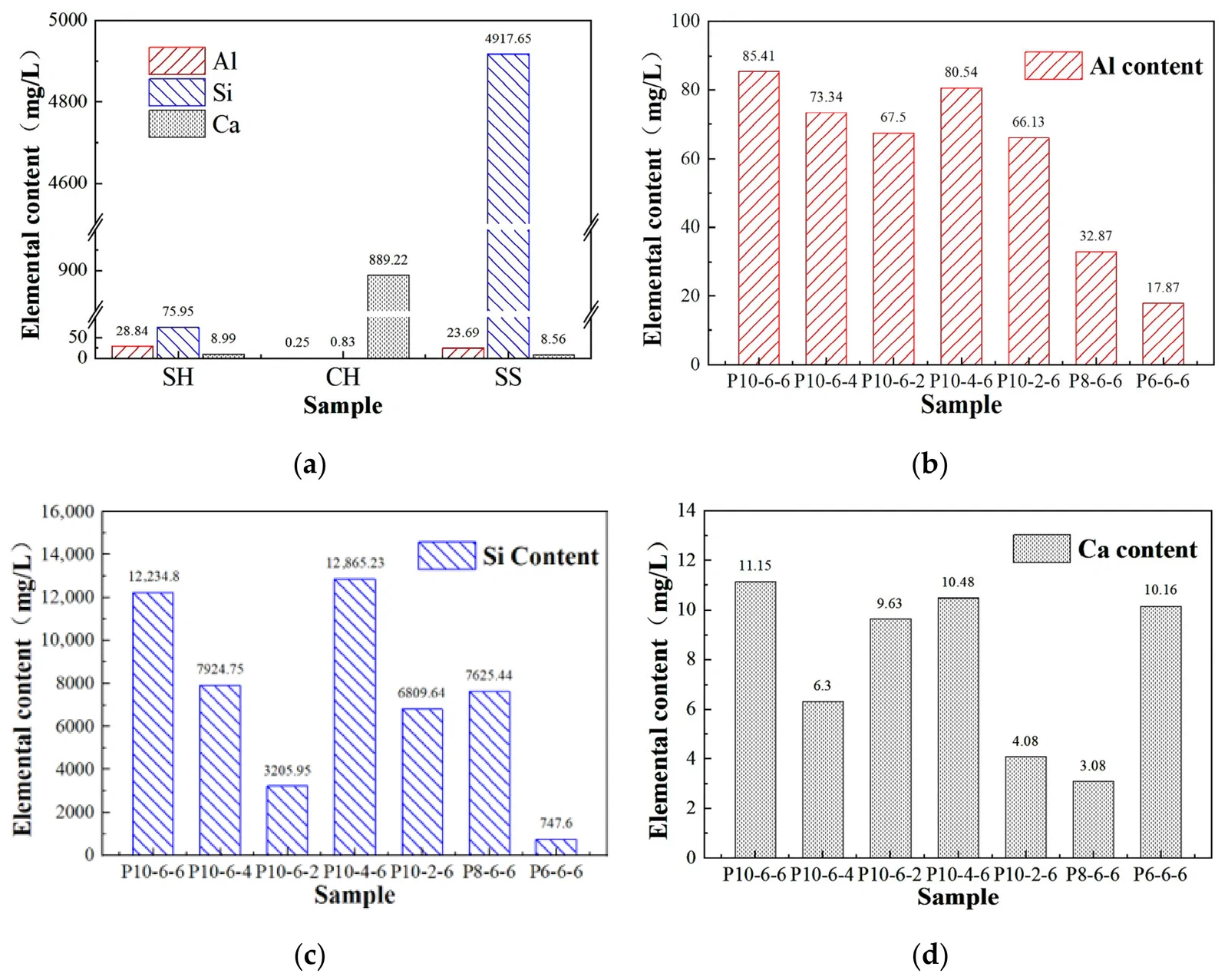
ICP-AES results. (**a**) Measured elemental concentrations under unitary alkali activation; (**b**) Measured Al concentrations under ternary composite alkali activation; (**c**) Measured Si concentrations under ternary composite alkali activation; (**d**) Measured Ca concentrations under ternary composite alkali activation.

Figure 2 presents the measured elemental concentrations in the unitary and ternary alkali-activation systems. As shown in Figure 2a, the peak measured Si and Ca concentrations were 4917.65 mg/L and 889.2 mg/L, respectively. Compared with SS treatment, SH treatment produced 21.7% and 5.02% higher measured Al and Ca concentrations, respectively, suggesting a stronger effect of SH on the measured elemental concentrations under the tested conditions. In contrast, owing to its low solubility, CH did not establish a highly alkaline environment, and the measured Si and Al concentrations were only 0.83 mg/L and 0.25 mg/L, respectively—markedly lower than those obtained with SH and SS. These findings indicate that, at equivalent dosages, the apparent elemental-release response associated with the three unitary alkali activators generally followed the order SH > SS > CH under the tested conditions. This trend may be associated with the high concentration of OH^−^ provided by SH, which can promote the reaction of mineral phases within the simulated lunar soil particles. Further analysis revealed that the measured Ca concentration in SH/SS systems was lower than the corresponding Si and Al concentrations (except in the CH system), primarily constrained by two factors: the low CaO content (<9 wt.%) in the simulated lunar soil matrix, and the homoionic effect, where CaO, as an alkaline earth oxide, may exhibit lower Ca release under the high alkalinity induced by SH/SS.

Figure 2b presents the measured Al concentrations in the ternary composite alkali-activation system. In the absence of an exogenous Al source, the measured Al concentration generally decreased as the alkalinity of the system was reduced. When the SS and CH dosages were reduced from 6 wt.% to 2 wt.%, the measured Al concentrations decreased to 79.0% and 77.4% of the corresponding reference values, respectively. By contrast, reducing the SH dosage from 10 wt.% to 8 wt.% and 6 wt.% decreased the measured Al concentration to 38.5% and 20.9% of the reference value, respectively, suggesting that SH dosage strongly influenced the availability of Al-containing species under the tested conditions, consistent with previous observations [32].

Figure 2c presents the measured Si concentrations in the ternary composite alkali-activation system. The effect of SH dosage on the measured Si concentration was similar to that observed for Al. Reducing the SH dosage from 10 wt.% to 8 wt.% and 6 wt.% decreased the measured Si concentration to 62.3% and 6.1% of the reference value, respectively, suggesting that the SH dosage strongly affected the availability of Si-containing species under the tested conditions. The measured Si concentration reflected both the direct contribution of Si from SS and the release of Si-containing species from the simulated lunar soil under alkaline conditions. As the SS dosage decreased from 6 wt.% to 4 wt.% and 2 wt.%, theoretical Si concentrations should reduce to 66.7% and 33.3% of the 6 wt.% level. However, the measured Si concentrations in the P10-6-6, P10-6-4, and P10-6-2 groups decreased to 64.8% and 26.2% of the P10-6-6 level—below the theoretical expectations (66.7% and 33.3%). This result suggests that the SS dosage influenced the measured Si concentration through both its direct Si contribution and its effects on the reactions occurring in the alkaline system. Reducing the CH dosage from 6 wt.% to 4 wt.% slightly increased the Si concentration due to partial dissociation of trace CaSiO_3_ precipitates resulting from the lowered Ca^2+^ concentration. A further reduction to 2 wt.% was associated with a lower measured Si concentration, possibly because the reduced alkalinity limited precursor reaction.

Figure 2d presents the measured Ca concentrations in the ternary composite alkali-activation system. Compared to unitary CH-activation, the measured Ca concentration in the ternary system was noticeably lower (Figure 2a,d) than in unitary CH-activation but higher than in SH- or SS-activation. This behavior may be associated with the influence of system alkalinity on CH dissolution and the subsequent reactions of dissolved Ca. The measured Ca concentration may reflect the combined effects of CH dissolution, Ca release from the precursor, and the consumption of dissolved Ca during the formation of Ca-bearing reaction products. Reducing the SH dosage from 10 wt.% initially decreased and then increased the measured Ca concentration: at 8 wt.% SH, the reduced precursor reaction may have lowered the Ca concentration, whereas at 6 wt.% SH, the lower alkalinity may have facilitated CH dissolution and Ca release. A similar pattern was observed for SS, suggesting that the measured Ca concentration was influenced by the alkalinity and associated reactions within the system. Lowering CH dosage from 6 wt.% to 4 wt.% resulted in a Ca concentration of 94.0% for the P10-6-6 group, while a further reduction to 2 wt.% decreased the measured concentration to 36.6%, following a trend similar to that observed for Si. This suggests that a CH dosage of ≥4 wt.% was associated with comparatively stable measured Ca/Si concentrations under the tested conditions.

### 3.2. Flowability Analysis

**Figure 3:**
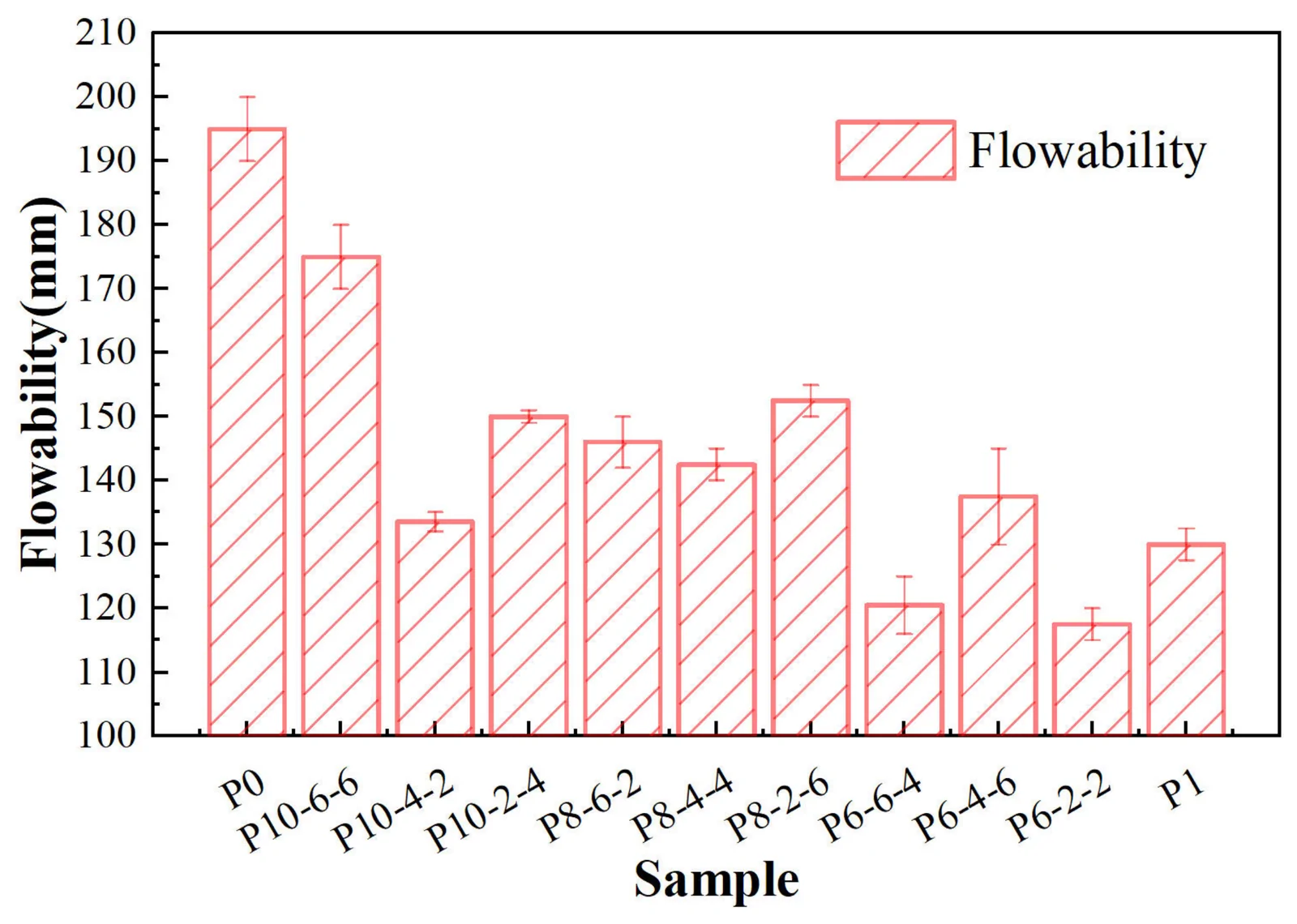
Flowability results of NS-SLSG.

Figure 3 illustrates the impact of composite alkali-activator formulations on the flowability of NS-SLSG slurry. A decrease in total activator dosage from 22 wt.% to 10 wt.% resulted in a 32.9% reduction in flowability. This decrease is due to higher alkalinity promoting dissolution of coarse precursor particles, yielding refined slurry fluidity; lower alkali dosage weakened simulated lunar soil dissolution, impairing flowability. This indicates appropriate alkali dosage critically maintains NS-SLSG workability, consistent with Zhong’s results [33].

Range analysis (*Ri*) was used for the orthogonal tests [34] as a descriptive method to compare the relative magnitude of the effects of different factors: a larger *Ri* indicates a greater variation in the response across the investigated factor levels rather than statistical significance. *Kn* represents the average result at factor level n, with *Ri* = max(*Kn*) − min(*Kn*). Table 3 presents the range analysis results for NS-SLSG flowability. The primary-to-secondary influence order is SH > SS > CH, with *Ri* values of SH and SS being 2.96 and 2.43 times CH’s, respectively, indicating their regulatory role in slurry flowability. The high alkalinity of SH significantly enhances dissolution of lunar soil, improving NS-SLSG flowability, as discussed in Section 3.1. In contrast, SS exhibits weaker dissolution capacity and slightly lower flowability influence than SH, while CH shows minimal impact due to low solubility. In the 16 wt.% alkali system, P8-2-6 formulation achieved optimal NS-SLSG flowability at 152.5 mm—only 12.9% lower than P10-6-6—demonstrating precise flow regulation through formulation optimization.

Flowability of P8-6-2 was observed to exceed that of its NS-free reference group P1 by 12.3%, due to lower viscosity in the SLSG slurry under reduced alkali dosage, which negatively affects workability and flow capacity. Based on the mechanisms reported in previous studies, NS may contribute to the development of silicate networks through interactions involving surface hydroxyl groups and dissolved silicates, thereby improving slurry viscosity and workability. In addition, possible filling and lubrication effects of NS may contribute to the improved flowability [35], while its dissolution under strongly alkaline conditions may provide additional reactive silicate species. This aligns with Zhang et al.’s findings [36] that sub-1.0% NS dosage enhances slurry flowability.

### 3.3. Mechanical Property

#### 3.3.1. Compressive Strength

**Figure 4:**
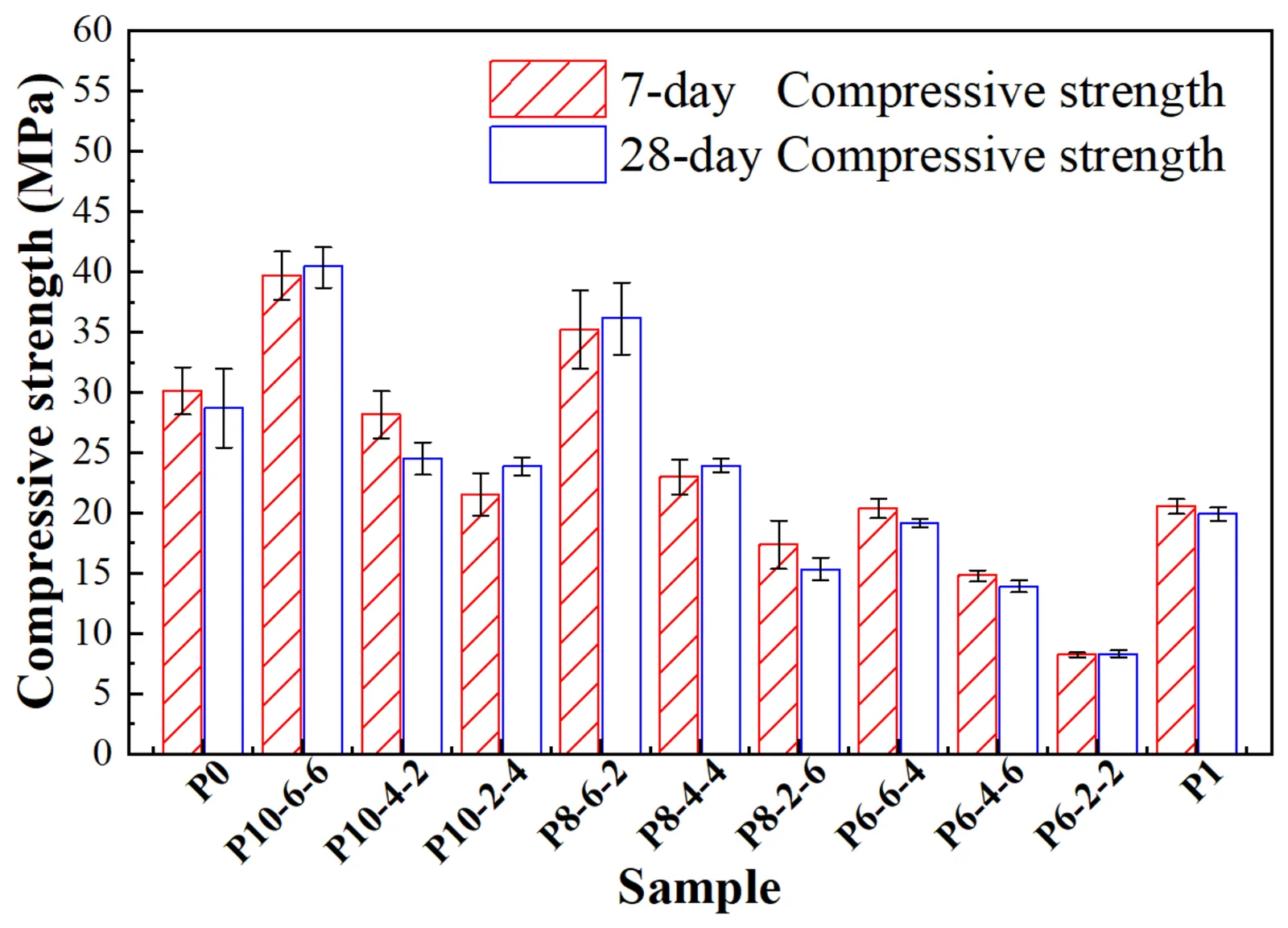
Compressive strength test results.

Figure 4 shows the compressive strength of NS-SLSG under different composite alkali-activator formulations. The activator concentration primarily affected the compressive strength. The maximum 28-day strengths recorded at total dosages of 22 wt.%, 16 wt.%, and 10 wt.% were 40.41 MPa (P10-6-6) [23], 36.15 MPa (P8-6-2), and 8.29 MPa (P6-2-2), respectively. High-concentration alkaline environments promote Si/Al dissolution from simulated lunar soil, accelerating condensation of monomers (Si(OH)_4_, Al(OH)_4_^−^) forming 3D networks for higher strength, aligning with Shahnazi et al. [37]. Even at identical 16 wt.% total dosage, compressive strengths varied significantly, with 28-day values ranging from 13.88–36.15 MPa. At ≤16 wt.% dosage, P8-6-2 exhibited the highest strength with 7-/28-day values of 35.23/36.15 MPa. With a 23.9% lower total admixture mass than the P0 group, it still achieved 26.0% higher 28-day strength via NS enhancement, demonstrating that NS effectively compensates for performance loss from reduced activator dosage. P10-6-6 showed an increase of 31.7%/40.8% in 7-/28-day strength over P0, while P8-6-2 achieved 71.5%/81.6% increases over P1, confirming better NS enhancement at low activator dosage consistent with Xu et al. [38]. The underlying mechanism for this performance improvement can be explained as follows. The P1 reference group exhibited poor flowability, which led to the formation of numerous curing defects [39], while the incorporation of NS significantly improved flowability, resulting in more uniform molding. The ICP-AES results (Section 3.1) showed that reducing the composite alkali-activator dosage was accompanied by lower measured Si and Al concentrations, suggesting a reduced availability of reactive Si- and Al-containing species for geopolymerization. According to previous studies, the high specific surface area and surface energy of NS may favor its pozzolanic reaction [40], while its surface Si–OH groups may form hydrogen bonds [41] and participate in polycondensation with Al(OH)_4_^−^ species derived from the depolymerization of the simulated lunar soil [42]. Under the strongly alkaline conditions and 100 °C curing regime used in this study, NS may also dissolve and act as an additional source of reactive silicate species. These possible effects may collectively contribute to gel-network formation under low-alkali conditions, thereby improving the mechanical properties of SLSG.

The P6-2-2 group demonstrated a 28-day compressive strength of 8.29 MPa with 10 wt.% activator dosage. This aligns with the 5–10 MPa threshold [43] for lunar-surface road materials, indicating high application potential for non-load-bearing structures. The higher compressive strengths of P10-6-6 and P8-6-2 indicate their potential for future load-bearing applications in lunar construction.

**Figure 5:**
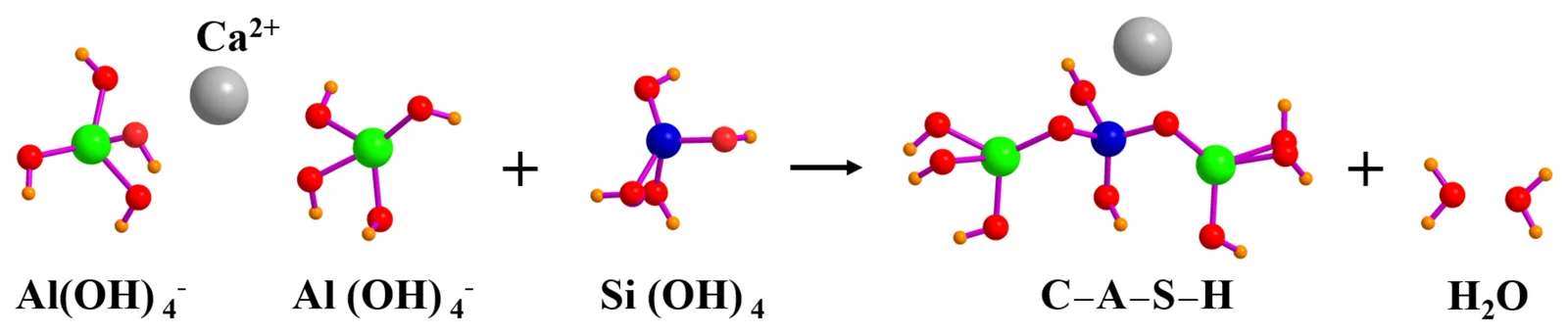
Schematic of Ca^2+^-enhanced polycondensation.

Table 4 presents Ri values for the 28-day compressive strengths of NS-SLSG, ranked as CH > SH > SS. Ri values for CH and SH are 18.08 and 17.76 times those of SS, indicating that SS has a noticeably smaller influence on compressive strength than CH and SH. Mechanistically, Ca^2+^ promotes slurry hardening and enhances viscosity [44], increasing gel products via a water-retention mechanism; CH releases more Ca^2+^, which has a stronger charge neutralization effect than Na^+^, electrostatically attracting Al(OH)_4_^−^ and condensing with Si(OH)_4_ via a nucleation effect to form C-A-S-H gel (Figure 5 illustrates CH-enhanced polycondensation). SH may promote the release of Si- and Al-containing species from simulated lunar soil particles by providing a highly alkaline environment, while its Na^+^ improves gel-network crosslinking via charge balance [45]. The comparatively weaker influence of SS may be related to the fact that all nine orthogonal mixtures contained 0.75 wt.% NS. The additional reactive silica supplied by NS may have brought the available silica content of the system closer to an appropriate level, thereby reducing the marginal response of compressive strength to variations in SS dosage. This interpretation is also consistent with the findings reported by Zhou et al. [46]. In addition, Wu et al. [14] reported that SS exhibited a more pronounced activation effect in precursors with a relatively low vitreous-phase content. Because TJ-1 contains more than 30% vitreous phase, this particular advantage of SS may be less evident in the present system. Further analysis reveals that the dosage levels corresponding to the optimal compressive strength for all three alkali activators are the high dosage levels, i.e., their initial levels. This trend supports the beneficial effect of relatively high activator dosages on the reaction of simulated lunar soil. The 28-day compressive strength of P8-6-2 reached 36.15 MPa, which was only 10.5% lower than that of P10-6-6. Compared with P0, P8-6-2 had a 23.9% lower total admixture mass while exhibiting a 26.0% higher 28-day compressive strength. This demonstrates that the high reactivity of NS can effectively compensate for the insufficient activation capacity under low-alkali activator dosage conditions, offsetting the associated performance loss. Considering the substantial reduction in activator mass, the P8-6-2 formulation still demonstrates significant economic advantages for lunar-surface construction.

#### 3.3.2. Flexural Strength

**Figure 6:**
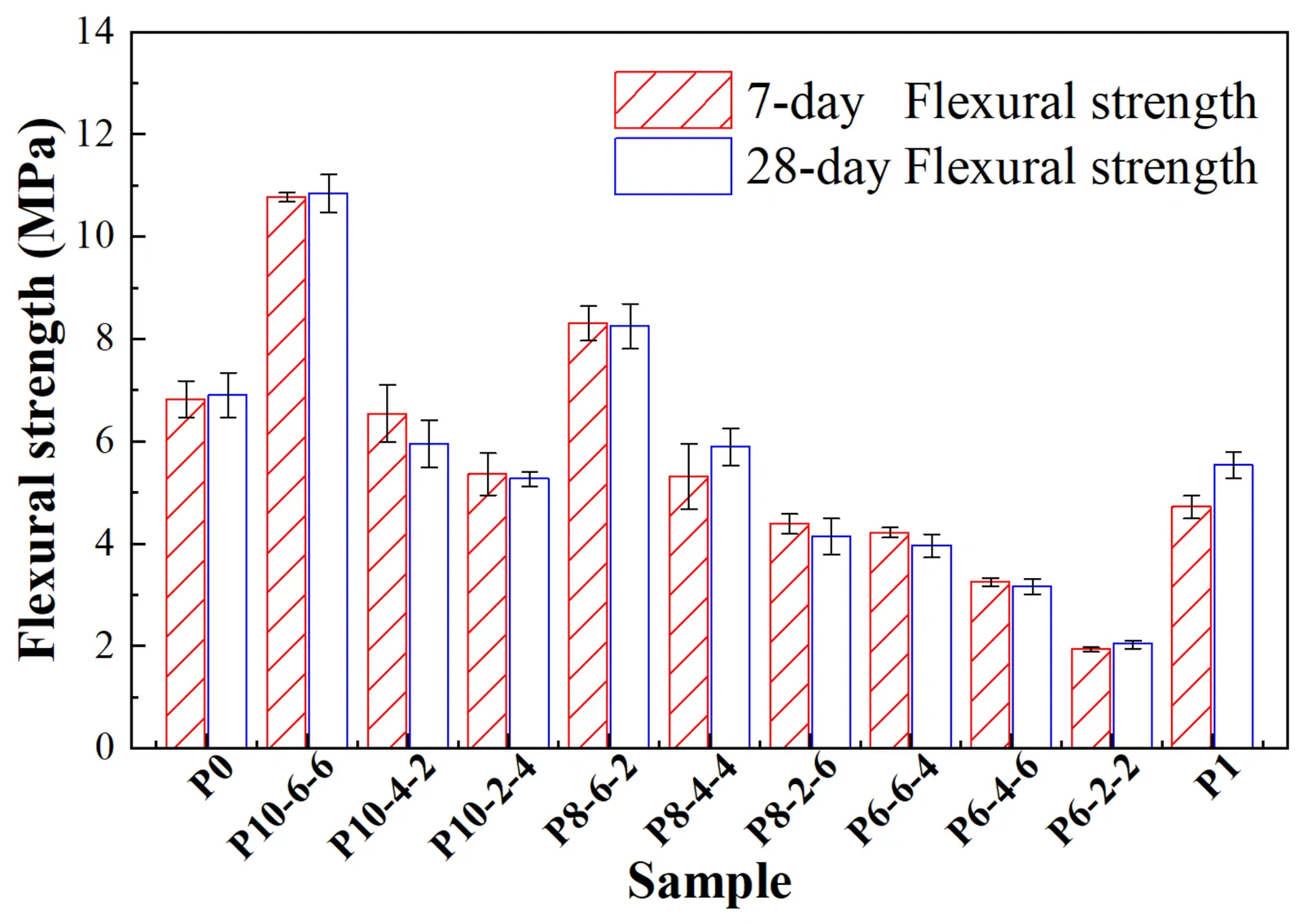
Flexural strength test results.

Figure 6 illustrates the flexural strength of NS-SLSG under varying composite alkali-activator formulations. Flexural strength declines with reduced activator dosage, mirroring compressive strength trends. Maximum 28-day strengths at 22 wt.%, 16 wt.%, and 10 wt.% total dosage were 10.85 MPa for P10-6-6 [23], 8.26 MPa for P8-6-2, and 2.04 MPa for P6-2-2.

At ≤16 wt.% dosage, 28-day flexural strength ranged from 2.04 to 8.26 MPa, with P8-6-2 exhibiting the highest values: 7-day 8.31 MPa and 28-day 8.26 MPa, showing 21.7% and 19.5% increases over the P0 group. This corroborates compressive strength results, confirming that NS reduces the required activator dosage. P10-6-6 achieved 57.7% and 57.0% 7-day and 28-day strength increases over P0, while P8-6-2 showed 76.1% and 49.0% increases over P1. 7-day enhancement of NS in P8-6-2 markedly exceeded that of P10-6-6 with comparable 28-day enhancement, further demonstrating superior NS effects at low activator dosage.

Table 5 presents Ri values for 28-day flexural strength of NS-SLSG. The influence order is SH > CH > SS, with Ri values of SH at 4.26 and CH at 3.83 times that of SS. This is different from compressive strength influences since flexural failure involves tension-compression stress fields, increasing sensitivity to internal defects like cracks/pores. Reduced SH dosage markedly decreases flowability, increasing curing-induced defects, while CH is weaker in influencing flowability. Reducing CH dosage does not noticeably increase curing defects in NS-SLSG; the efficacy of CH on flexural strength is slightly lower than that of SH. The influence of SS on flexural strength noticeably exceeds its effect on compressive strength (Table 5 and Section 3.3.1) due to improved flowability and reduced internal defects via enhanced slurry homogeneity. This indicates that SS and SH improve NS-SLSG flexural strength by modulating slurry flowability. Furthermore, it can be observed that the optimal levels for flexural strength of all three alkali activators also correspond to the high dosage levels, namely their initial levels, which is consistent with the compressive strength results. The combination of relatively high flexural strength and significantly reduced alkali-activator dosage in the P8-6-2 formulation further confirms its economic advantages for practical applications.

#### 3.3.3. Mass–Strength Efficiency

The resource utilization of NS-SLSG was analyzed using the mass–strength efficiency (MSE) evaluation system from Zhou et al. [20] for various composite alkali-activator formulations, as shown in Equation (1).

(1) $$\eta_{C/Fm}={\left[\frac{\left(m_{AE}+m_{NS}\right)\times 100}{m_{TJ}\times f}\right]}^{-1}$$

Nomenclature: $m_{AE}$: Mass of alkali activators (g); $m_{NS}$: Mass of NS (g); $m_{TJ}$: Mass of TJ-1-simulated lunar soil (g); *f*: Compressive or flexural strength (MPa); $\eta_{Cm}$, $\eta_{Fm}$: Compressive and flexural strength mass–strength efficiency.

**Figure 7:**
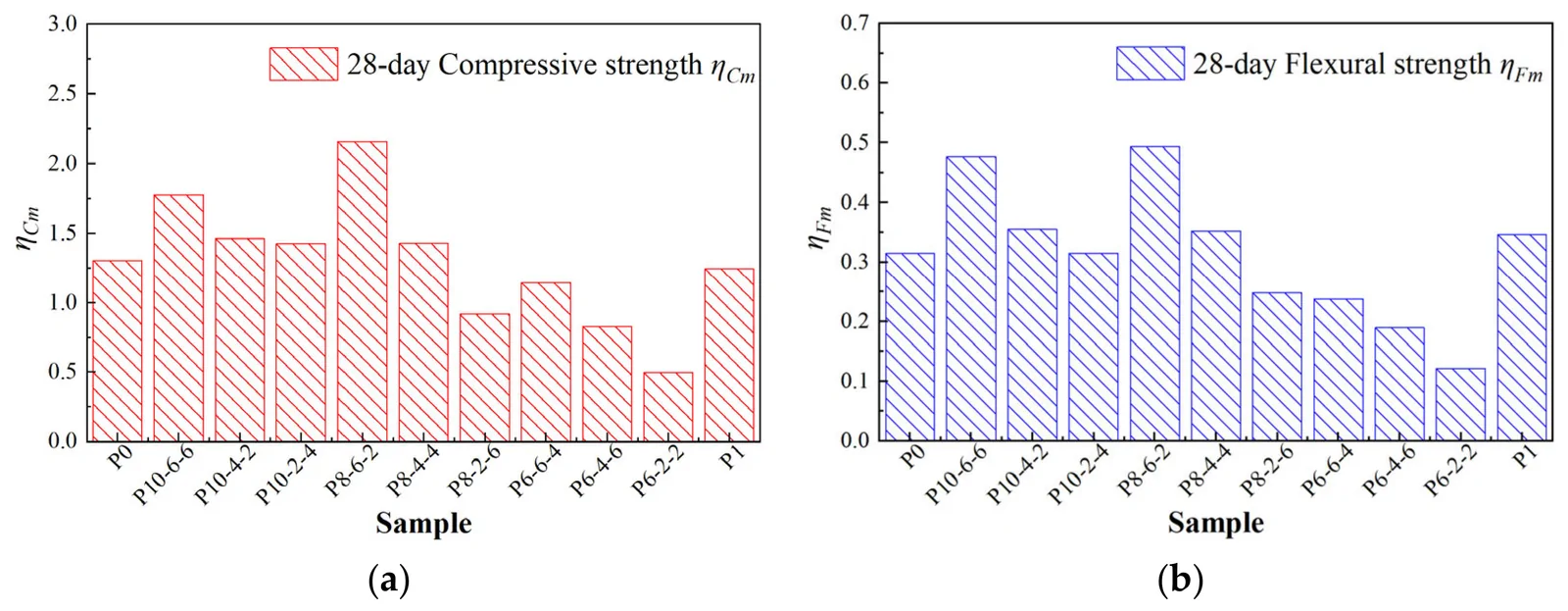
Mass–strength efficiency. (**a**) Compressive strength; (**b**) Flexural strength.

Figure 7a,b present the MSEs of 28-day compressive and flexural strengths of SLSG under varying composite alkali-activator ratios. Figure 7a shows that reducing the activator dosage generally decreases $\eta_{Cm}$ due to a reduction in mechanical properties; however, the P8-6-2 group achieves a significantly higher $\eta_{Cm}$ of 2.16, which is 66.2%, 21.4%, and 74.2% higher than that of the P0, P10-6-6 [23], and P1 groups, respectively, through reasonable regulation of the activator mixing ratio as in the P8-6-2 group. Figure 7b shows that the development pattern of $\eta_{Fm}$ of SLSG is similar to that of $\eta_{Cm}$; except for the P8-6-2 group, as the activator dosage decreases the $\eta_{Fm}$ with a gradually decreasing trend. $\eta_{Fm}$ for the P8-6-2 group is 0.49, which is 58.1%, 2.1%, and 40.0% higher than that of the P0, P10-6-6, and P1 groups, respectively. This indicates that reasonable regulation of activator dosage and mixing ratio helps improve the $\eta_{m}$ of SLSG and reduces the admixture dosage required for lunar soil solidification to achieve unit strength (1 MPa).

The $\eta_{Cm}$ for the P1 group is 4.6% lower than that of the P0 group, indicating that reducing the composite alkali-activator dosage deteriorates mechanical properties and resource utilization efficiency. By enhancing mechanical properties and supplementing the silicon source, NS enabled the P8-6-2 group to reduce the admixture dosage required per unit compressive and flexural strength by 74.2% and 40.0%, respectively, compared with the P1 group.

### 3.4. Microstructure

#### 3.4.1. FT-IR Analysis

**Figure 8:**
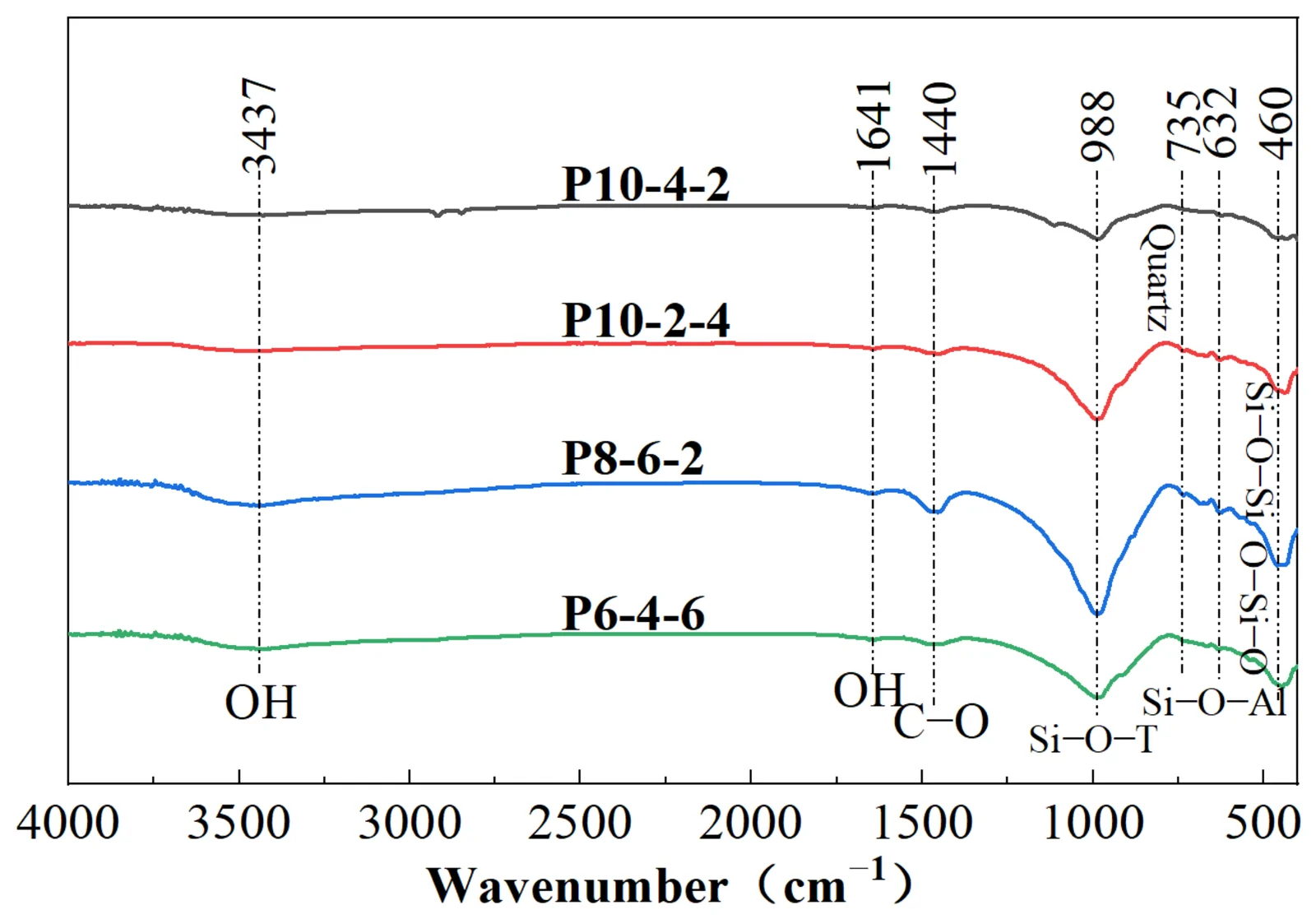
FT-IR spectra of NS-SLSG.

Figure 8 shows the FT-IR test results for the P10-4-2, P10-2-4, P8-6-2, and P6-4-6 groups. As evident in Figure 8, NS-SLSG with varying composite activator ratios exhibits the same major chemical bond types, with the main absorption peak at 988 cm^−1^ originating from asymmetric stretching vibration of Si–O–T bonds (T represents Si or Al) [47,48]. This band is commonly associated with Si–O–T environments in aluminosilicate reaction products, including N-A-S-H- and C-A-S-H-type gels. The relatively weaker main absorption band of the P10-4-2 group may be related to its lower SS dosage and the corresponding reduction in silicate species contributing to the Si–O–T bonding environment. The P10-2-4 group displays a higher main absorption peak intensity than the P10-4-2 group; however, its mechanical properties are inferior, as higher SS dosage introduces more Si-O-Si while lower CH dosage reduces Ca^2+^ concentration, limiting polycondensation and decreasing the degree. The P8-6-2 group exhibited the strongest Si–O–T absorption band, which was consistent with a more extensively developed aluminosilicate reaction-product network and may be associated with the combined effects of SH and CH on precursor reaction and reaction-product formation. Despite having the highest SS dosage, the P6-4-6 group exhibited a weaker Si–O–T absorption band than the P8-6-2 group, which may be associated with the lower SH dosage and the correspondingly reduced availability of reactive aluminosilicate species.

The absorption bands in the range of 600–800 cm^−1^ were assigned to Si–O–Al bending vibrations [49,50], supporting the presence of Al-containing aluminosilicate structures and indicating that the activator ratios influenced their local bonding environments. The bands near 3437 and 1641 cm^−1^ were assigned to O–H stretching and H–O–H bending vibrations, respectively [51]. Their higher intensities in the P8-6-2 group may reflect differences in hydroxyl-containing reaction products and water-binding environments, consistent with its comparatively high mechanical properties. In addition, an absorption band at approximately 1440 cm^−1^ was observed in all specimens and was assigned to the asymmetric C–O stretching vibration of carbonate groups [52]. This band may be attributed to partial carbonation caused by the ingress of CO_2_ from the surrounding air during curing under atmospheric pressure.

#### 3.4.2. XRD Analysis

**Figure 9:**
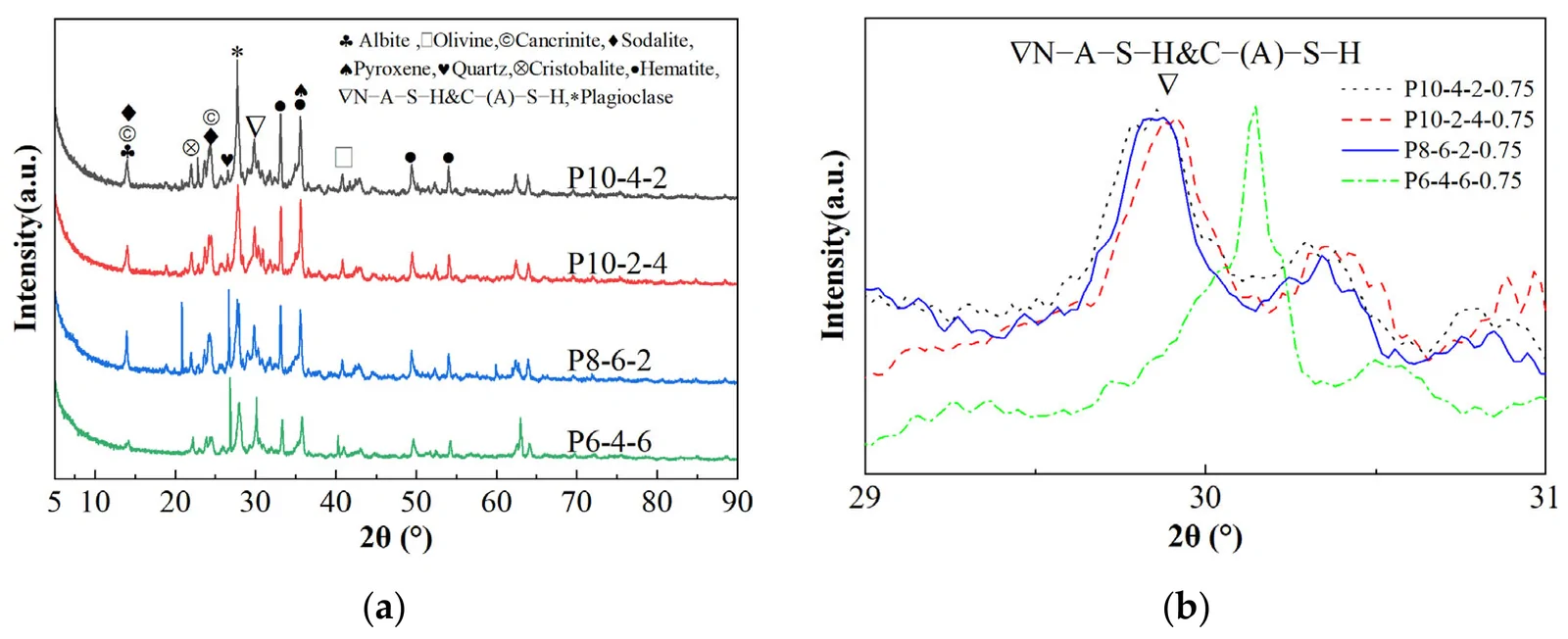
XRD patterns of NS-SLSG. (**a**) XRD pattern of the sample; (**b**) Enlarged section of the XRD pattern.

Figure 9 shows the XRD patterns of the P10-4-2, P10-2-4, P8-6-2, and P6-4-6 groups. The NS-SLSG specimens prepared with different composite activator ratios exhibited generally similar phase compositions, mainly including olivine, albite, pyroxene, zeolitic phases such as sodalite and cancrinite that may form under alkaline conditions, and other minor crystalline phases. Owing to the complex multiphase composition of simulated lunar soil and its alkali-activated products, some diffraction peaks may overlap. Specifically, the diffraction feature at approximately 14° may contain overlapping contributions from albite, cancrinite, and a sodalite-type phase; the diffraction feature near 22° is broadly consistent with the main reflection of cristobalite; and the strong diffraction peak near 35.6° may mainly contain contributions from pyroxene and hematite.

Figure 9a indicates that different composite activator ratios affect the depolymerization and polycondensation processes of NS-SLSG, resulting in differences in its characteristic diffraction peaks. The characteristic peaks of quartz appear at approximately 26.7–26.9° (2θ) in the P8-6-2 and P6-4-6 groups, which may be associated with the limited dissolution of quartz in the simulated lunar soil at relatively low SH dosages [53,54]. The P8-6-2 group exhibits a strong diffraction peak at approximately 20.8–21.0° (2θ), which may originate from quartz or may be associated with a pre-peak of C-A-S-H gel [55]. No obvious corresponding peak is observed in the P6-4-6 group at the same position. Therefore, this diffraction feature in the P8-6-2 group may not be attributed entirely to quartz and may also contain a contribution from the C-A-S-H gel pre-peak.

Figure 9b shows that all test groups exhibit a distinct broad diffraction feature at approximately 30.0° (2θ), which is commonly associated with the formation of N-A-S-H and C-(A)-S-H gels [56]. The P6-4-6 group exhibits relatively sharper diffraction peaks with a certain tendency to shift toward higher angles. This may be because the weaker alkaline environment restricts Al dissolution from the simulated lunar soil, resulting in a relatively higher Si/Al ratio and enhancing the contribution of Si–O bonds to the gel network, which may decrease the interplanar spacing and shift the diffraction peaks toward higher angles. The P6-4-6 group contains less Na^+^, while Al dissolution is restricted, which may relatively favor the formation of C-S-H gel. C-S-H gel may possess relatively more ordered local layered or spherical structures, thereby contributing to the sharper diffraction features [57]. Nevertheless, differences in relative phase content and diffraction-peak overlap may also affect the peak profiles and positions; therefore, the above analysis is mainly used to explain the relative variations among the different specimens.

In addition, diffraction-peak broadening may provide some indication of the presence of poorly crystalline phases or small coherent diffraction domains. Avram et al. [58] calculated a kaolinite coherent-domain size of approximately 68 nm using the Scherrer equation based on its broadened diffraction peaks and obtained a particle size of approximately 75 nm through AFM observations. Their study indicates that combining XRD peak-width analysis with high-resolution microscopic observations can be used to discuss nanometric mineral particles. In the present study, the broad gel-related diffraction feature near 30° and several broadened crystalline reflections may be associated with the formation of poorly crystalline and finely distributed reaction products. However, considering that peak overlap, microstrain, and instrumental broadening may also affect the measured diffraction-peak widths, the present XRD results provide only qualitative evidence for the presence of fine or poorly crystalline reaction products and cannot directly determine their actual particle sizes.

#### 3.4.3. TG-DSC-DTG Analysis

**Figure 10:**
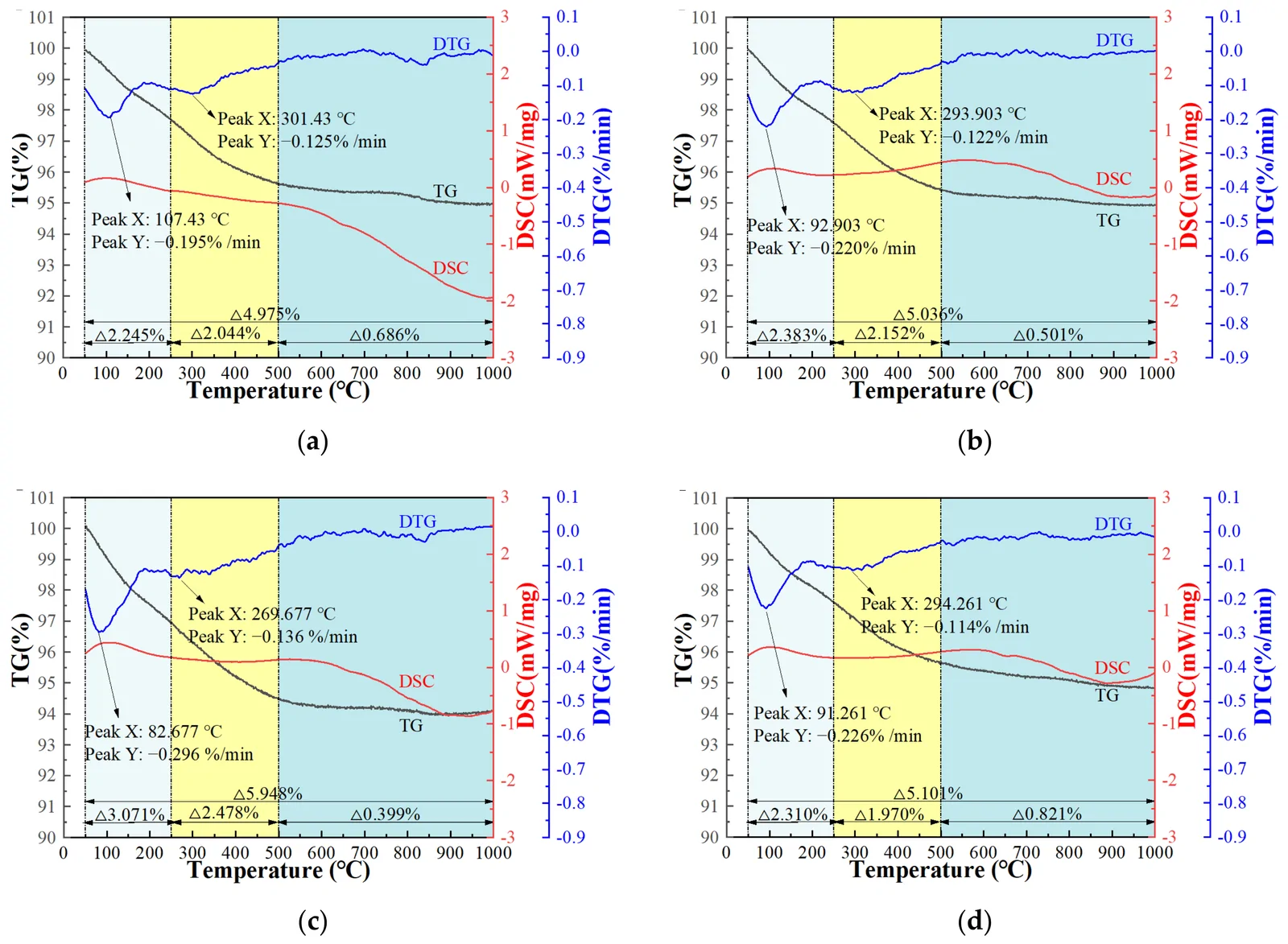
TG-DSC-DTG curves of NS-SLSG; (**a**) P10-4-2; (**b**) P10-2-4; (**c**) P8-6-2; (**d**) P6-4-6.

Figure 10 presents the TG-DSC-DTG test results for the P10-4-2, P10-2-4, P8-6-2, and P6-4-6 groups. The TG curve and DSC curve are the mass-loss curve and the endothermic/exothermic curve of NS-SLSG with temperature, and the DTG curve is the first derivative of the TG curve, reflecting the mass-loss rate of NS-SLSG with temperature. Based on thermal decomposition characteristics, the temperature interval is divided into three regions: A (50–250 °C), B (250–500 °C), and C (500–1000 °C).

Mass loss in region A mainly originated from evaporation of free water and desorption of some weakly bound water [59,60]. Figure 10 depicts the mass loss in region A for each test group, following the order P8-6-2 > P10-2-4 > P6-4-6 > P10-4-2. Mass loss in region A for the P8-6-2 group is 3.071%, which is 28.9%, 32.9%, and 36.8% larger than that of the P10-2-4, P6-4-6, and P10-4-2 groups, respectively. Higher CH dosage in the P8-6-2 group adsorbs more free water with high Ca content, promoting C-A-S-H gel formation and more weakly bound water, consistent with O–H absorption-band results (Section 3.4.1). DTG curves produce the most prominent peak in region A, indicating the highest mass-loss rate. The P10-4-2, P10-2-4, and P6-4-6 groups show closer mass-loss rates in the range 0.195–0.226%/min, while the P8-6-2 group exhibits a significantly higher mass-loss rate of 0.296%/min.

Mass loss in region B originates from the loss of chemically bound water and dehydroxylation of geopolymer gels [61,62], confirmed by endothermic peaks in DSC curves and mass-loss rate peaks in DTG curves. Mass loss in region B follows the order P8-6-2 > P10-2-4 > P10-4-2 > P6-4-6, with the P8-6-2 group exhibiting 2.478% mass loss in region B, which is 15.1%, 21.2%, and 25.8% higher than the P10-2-4, P10-4-2, and P6-4-6 groups, respectively, indicating higher gel content in the P8-6-2 group. This quantitatively confirms that CH enhances gel product formation in NS-SLSG by promoting the polycondensation reaction, consistent with the findings of Section 3.3. The P6-4-6 group shows the lowest mass loss in region B, aligning with its inferior mechanical properties due to insufficient SH dosage, hindering soil dissolution.

Mass loss in region C stems from viscous sintering and vitrification at high temperatures [63], significantly destabilizing the NS-SLSG structure. Mass-loss rates stabilize across groups in this region, indicating good thermal stability of NS-SLSG at elevated temperatures. Mass loss in region C follows the order P6-4-6 > P10-4-2 > P10-2-4 > P8-6-2 with P8-6-2 showing 0.399% mass loss, which is 51.4%, 41.8%, and 20.4% lower compared to the P6-4-6, P10-4-2, and P10-2-4 groups, respectively. This demonstrates better thermal stability of the P8-6-2 group, further confirming that rational regulation of composite alkali-activator dosage and ratio improves high-temperature thermal stability of NS-SLSG.

Compared with the P6-4-6 group, the P8-6-2 group exhibited 25.8% greater mass loss in region B and 51.4% lower mass loss in region C. These trends indicated that the composite alkali-activator ratio influenced both reaction-product development and the thermal response of NS-SLSG, which may have contributed to the observed differences in mechanical properties.

#### 3.4.4. SEM Analysis

**Figure 11:**
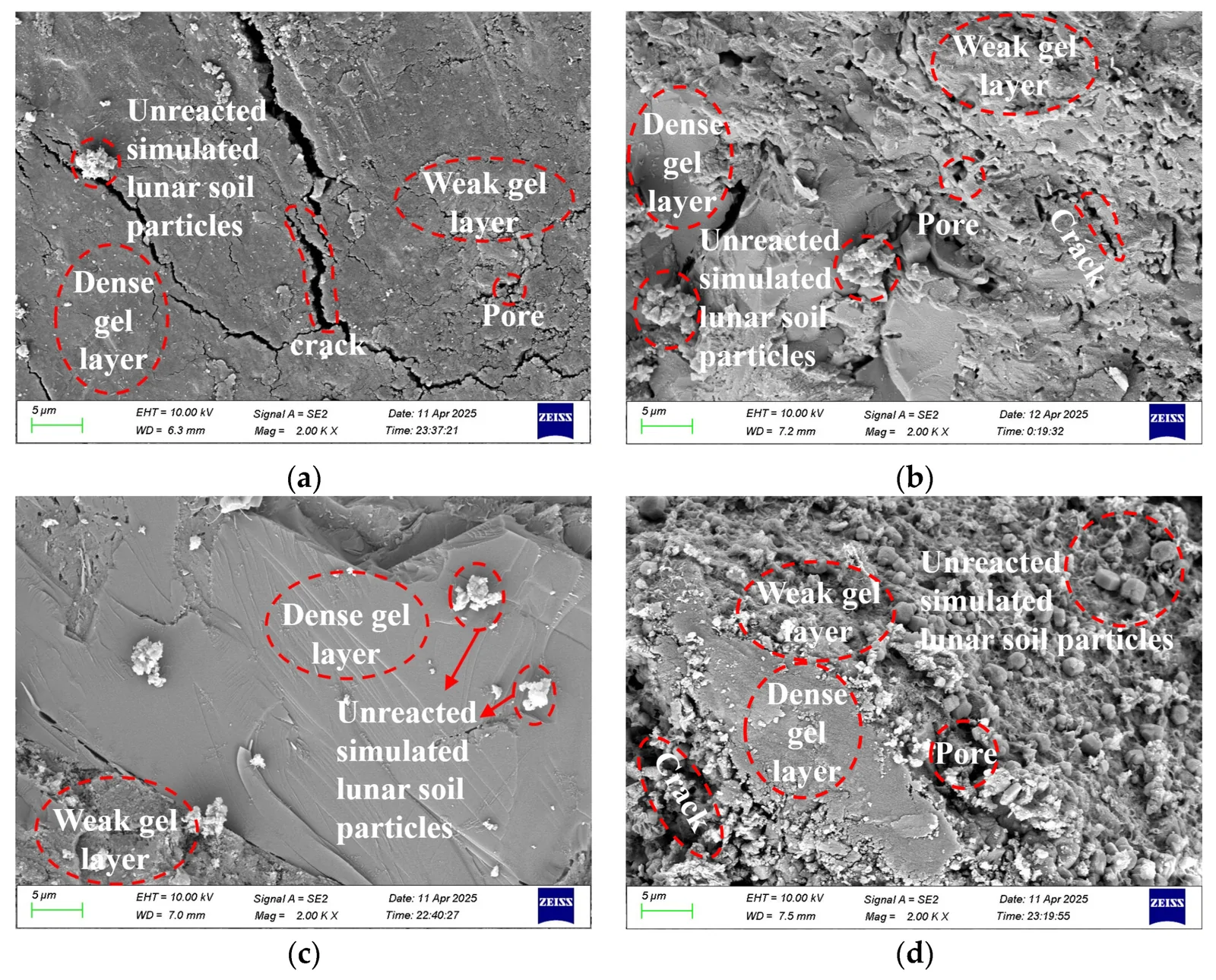
NS-SLSG magnified 2000× SEM images; (**a**) P10-4-2; (**b**) P10-2-4; (**c**) P8-6-2; (**d**) P6-4-6.

Figure 11 shows the 2000× SEM microscopic morphology of the P10-4-2, P10-2-4, P8-6-2, and P6-4-6 groups. The surface of the P10-4-2 group is relatively smooth, dominated by a dense gel layer; however, low CH content limits polycondensation, forming an incomplete structure with distributed through-cracks in the gel network. A few unreacted soil particles appear on the P10-4-2 surface due to high SH dosage, which enabled sufficient dissolution of the particles.

The P10-2-4 group contains more pores and microcracks (Figure 11b) with dense and weak gel layers distributed together, attributed to low CH dosage severely affecting polycondensation. A few unreacted soil particles on the P10-2-4 surface align with the P10-4-2 group, indicating that SH dosage dominates soil depolymerization, consistent with ICP-AES results (Section 3.1).

The P8-6-2 group exhibited a comparatively dense reaction-product structure with fewer visible pores and cracks (Figure 11c), consistent with its superior mechanical properties. This occurs because 8 wt.% SH enables sufficient soil dissolution, while a higher CH dosage promotes complete polycondensation, indicating that activator ratio selection should fully consider the depolymerization-polycondensation balance for better geopolymerization. The P8-6-2 surface shows more unreacted simulated lunar soil particles than those of the P10-4-2 and P10-2-4 groups since reduced SH dosage limits dissolution, while CH and SS exert weaker influence on depolymerization, consistent with ICP-AES in Section 3.1 and XRD results in Section 3.4.2.

The P6-4-6 group exhibits significantly different microscopic morphology (Figure 11d) from other groups, featuring an incomplete gel structure dominated by a weak gel layer with numerous pores and cracks distributed on the network due to low gel content, consistent with TG-DSC-DTG. The gel layer is covered with abundant unreacted simulated lunar soil particles, primarily indicating severely limited depolymerization from low SH dosage and compounded by low CH dosage, causing the poorest geopolymerization consistent with FT-IR and XRD results.

#### 3.4.5. EDS Analysis

**Figure 12:**
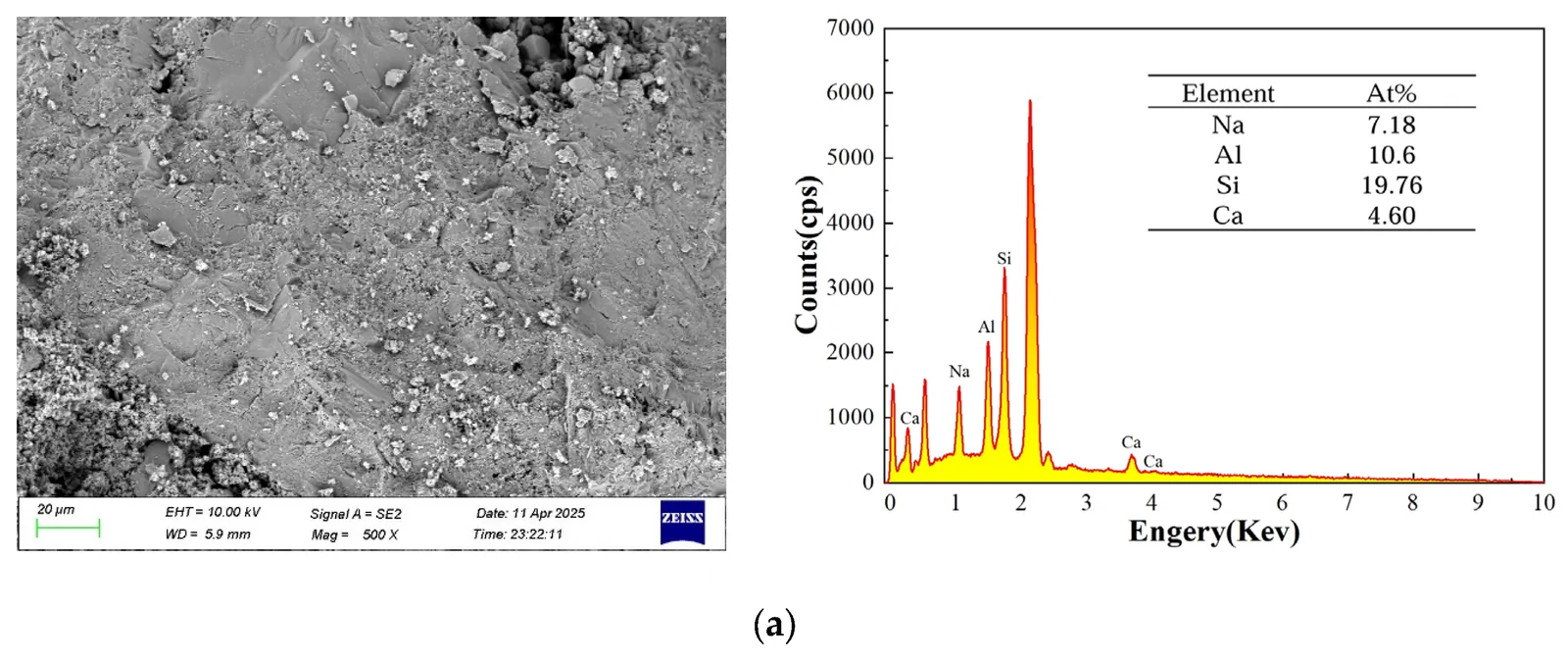

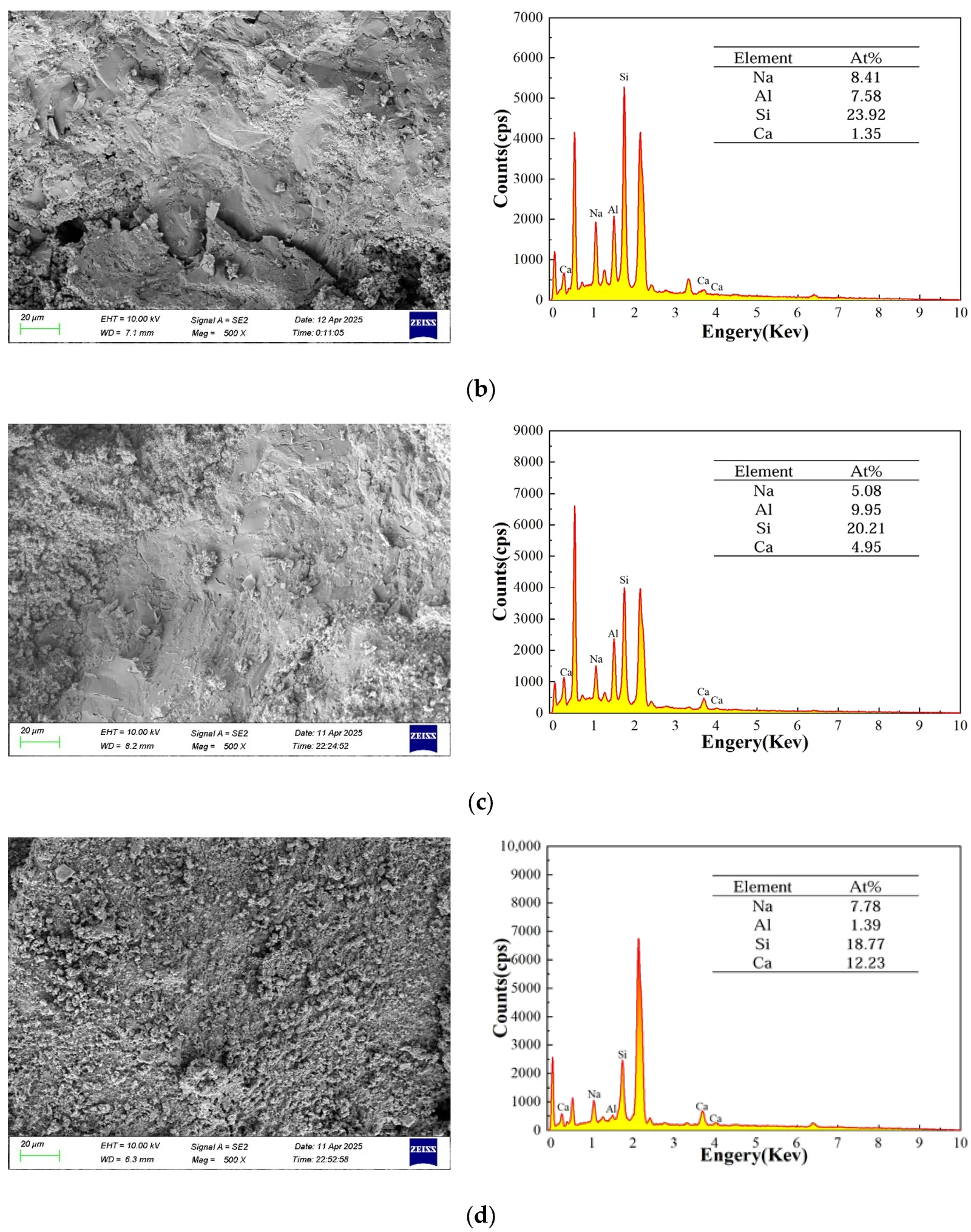
Typical EDS elemental test results; (**a**) P10-4-2; (**b**) P10-2-4; (**c**) P8-6-2; (**d**) P6-4-6.

Research shows that C-A-S-H gels accelerate slurry coagulation and confer higher early strength, while N-A-S-H gels offer better chemical stability and later strength [62,64]. To evaluate the gel chemical structure of NS-SLSG under different activator ratios, random sampling was taken from dense areas, ensuring at least 10 sampling points. EDS tests analyzed Na, Al, Si, and Ca, with averaged results for Al/Si, Na/Al, and Ca/Si atomic ratios. Figure 12 shows typical EDS results for each group, while Table 6 presents elemental contents and atomic ratios. The P10-4-2 group exhibits Al/Si, Na/Al, and Ca/Si ratios of 0.34, 0.63, and 0.24, respectively (Figure 12a and Table 6), indicating a balanced gel composition without dominant phase tendency.

As shown in Figure 12a,b and Table 6, the P10-2-4 group exhibited lower Na, Al, Si, and Ca contents than the P10-4-2 group. This may be attributed to its lower CH dosage, which limited Ca^2+^-assisted polycondensation and reduced the development of Ca-containing reaction products [65]. Its Ca/Si ratio was 70.8% lower, and its Na/Al ratio was 44.4% higher than those of the P10-4-2 group, indicating a shift toward a low-Ca, Na-rich local composition compatible with an N-A-S-H-type reaction-product environment [66].

The P8-6-2 group exhibited higher Na, Al, Si, and Ca contents than the P10-2-4 group (Figure 12b,c and Table 6). The higher CH dosage may have increased Ca^2+^ availability and facilitated Ca^2+^-assisted polycondensation, thereby promoting Ca incorporation into the reaction products. Consequently, its Ca/Si ratio was 343.9% higher, and its Na/Al ratio was 23.1% lower than those of the P10-2-4 group, indicating a shift toward a Ca-rich local composition compatible with C-A-S-H-type reaction products [67].

The P6-4-6 group exhibited lower local Si and Al contents than the other groups (Figure 12a–d and Table 6). This may be attributed to its lower SH dosage, which restricted precursor depolymerization and reduced the release of Si- and Al-containing species from the simulated lunar soil, consistent with the ICP-AES trends in Section 3.1. The P6-4-6 group exhibited a Ca/Si ratio of 0.92, which is compatible with the compositional range commonly reported for C-S-H-type reaction products (0.8–1.5) [68]. Its Al/Si ratio was 0.18, representing reductions of 47.1%, 18.2%, and 60.0% relative to the P10-4-2, P10-2-4, and P8-6-2 groups, respectively. The restricted release of Al-containing species, together with the Ca supplied by CH, may have favored the development of a comparatively Ca- and Si-rich C-S-H-type reaction-product environment. This compositional tendency was consistent with the sharper and shifted diffraction features observed in Section 3.4.2.

Different composite alkali-activator ratios were associated with distinct local elemental compositions in the dense reaction-product regions of NS-SLSG. The results suggest that SH promoted precursor depolymerization by providing a highly alkaline environment, thereby increasing the availability of dissolved Si- and Al-containing species and contributing Na^+^ to Na-rich aluminosilicate reaction products. CH supplied Ca^2+^ and facilitated Ca^2+^-assisted polycondensation, promoting the development of Ca-rich C-A-S-H-type reaction products. SS provided supplementary Si, which may have combined with the Ca^2+^ supplied by CH to facilitate the development of Si- and Ca-rich C-S-H-type reaction products. Consequently, variations in the SH, CH, and SS dosages influenced the local reaction-product composition of NS-SLSG through their respective effects on precursor depolymerization, Ca^2+^-assisted polycondensation, and Si supply.

#### 3.4.6. MIP Analysis

**Figure 13:**
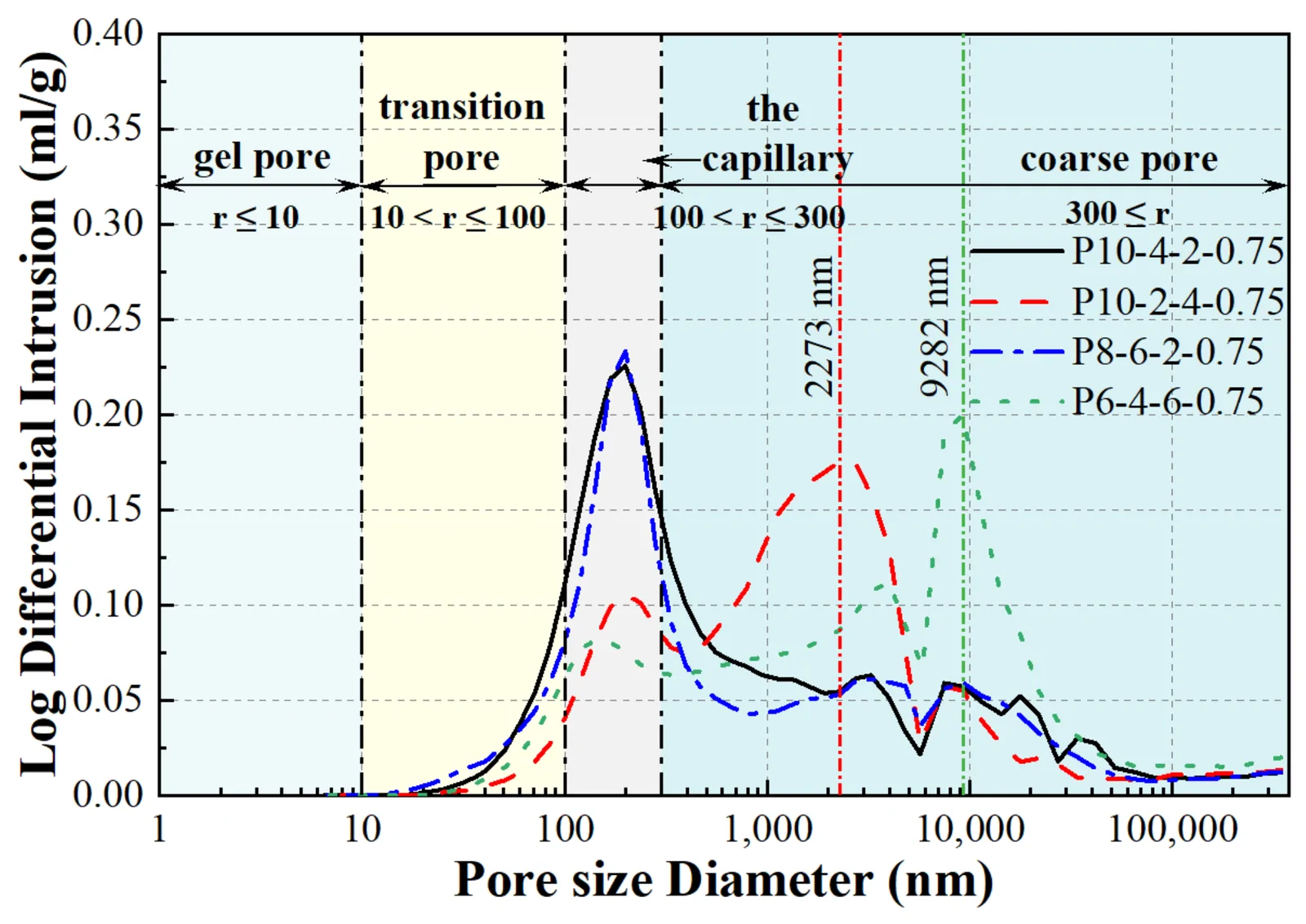
Cumulative pore volume distribution curves of NS-SLSG specimens.

Figure 13 depicts the cumulative pore volume distribution curves for the P10-4-2, P10-2-4, P8-6-2, and P6-4-6 groups. According to pore size classification criteria [69], pores can be categorized as gel, transition, capillary, and coarse. Figure 13 illustrates that the P10-4-2 group shows a primary distribution in the capillary pore range and lower coarse pore content, indicating that its pore forms mainly from water evaporation at high temperatures. The P10-2-4 group shows the main pore distribution within the coarse pore range with a coarse pore peak value of 2273 nm, resulting in reduced mechanical properties compared to the P10-4-2 group. The P8-6-2 group demonstrates pore characteristics similar to the P10-4-2 group, with the main pore size distribution in the capillary pore range, while coarse pore content further decreases compared to the P10-4-2 group; thus, the P8-6-2 group exhibits better mechanical properties than the P10-4-2 group. The P6-4-6 group displays a pore structure resembling the P10-2-4 group, dominated by coarse pores with a higher coarse pore peak value of 9282 nm, attributed to increased curing defects from insufficient slurry fluidity combined with insufficient geopolymerization degree, preventing the gel phase from adequately filling pore structures.

Table 7 shows the pore-structure parameters for the P10-4-2, P10-2-4, P8-6-2, and P6-4-6 groups. The P8-6-2 group has 35.88% porosity (Table 7), representing 8.7%, 8.0%, and 9.9% reduction compared to the P10-4-2, P10-2-4, and P6-4-6 groups, respectively. This demonstrates that higher CH dosage increases the geopolymerization rate and generates more C-(A)-S-H gels through Ca^2+^ introduction, contributing to a denser structure, to some extent consistent with findings of Lv et al. [70].

The P8-6-2 group exhibits the lowest average pore size, optimal probable pore size, and median pore size. The P10-2-4 and P6-4-6 groups show significantly higher mean and optimal probable pore sizes than the P10-4-2 and P8-6-2 groups due to the predominance of coarse pores in their pore structures. The P10-4-2 and P10-2-4 groups have median pore sizes of 135.09 nm and 178.54 nm, respectively, higher than 127.42 nm for the P6-4-6 group, yet demonstrate superior mechanical properties. A combined analysis with Figure 13 reveals that this occurs because the P6-4-6 group has significantly larger coarse pore sizes and higher coarse pore content than the P10-4-2 and P10-2-4 groups, leading to inferior mechanical properties.

## 4. Discussion

### 4.1. Preliminary Economic and Resource-Efficiency Analysis

The results of this study demonstrate that NS exhibits a more pronounced enhancement effect on SLSG at lower alkali-activator dosages, which is consistent with the observations of Xu et al. [38]. Leveraging this property, the incorporation of 0.75 wt.% NS effectively compensated for the insufficient activation efficiency at reduced alkali levels, enabling a reduction in the total admixture content from 22.00 wt.% to 16.75 wt.%, corresponding to a 23.9% reduction in the total admixture mass in the experimental formulation, while still achieving 26.0% and 19.5% increases in compressive and flexural strengths, respectively, compared to the P0 group. In terms of mass–strength efficiency, the P8-6-2 group achieved compressive and flexural mass–strength efficiencies of 2.16 and 0.49, respectively, corresponding to 74.2% and 40.0% reductions in admixture dosage required per unit strength relative to the P1 group.

To further evaluate the economic implications of future raw-material supply arrangements, this section adopts an anhydrous SS transportation scenario. The SS used experimentally was Na_2_SiO_3_·9H_2_O, which contains approximately 57.05 wt.% water of crystallization. Since crystallization water increases the Earth–Moon transportation mass, anhydrous SS with an equivalent silicate modulus should preferably be used for future transportation, with the corresponding water supplied on the lunar surface. Accordingly, the following transportation-mass and cost calculations use anhydrous-equivalent SS masses while maintaining the nominal Na_2_O and SiO_2_ inputs of the original experimental formulations. This conversion applies to the economic analysis of a future supply scenario; the experimental formulations and measured properties remain based on the materials actually used.

Per kilogram of dry simulated lunar soil, the SS masses in the P0 and P8-6-2 experimental formulations were 0.060 kg and 0.020 kg, corresponding to anhydrous SS masses of 0.02577 kg and 0.00859 kg, respectively. Under the anhydrous transportation scenario, the total transported admixture mass for P0 was therefore 0.18577 kg, comprising 0.100 kg SH, 0.060 kg CH, and 0.02577 kg anhydrous SS. The corresponding mass for P8-6-2 was 0.15609 kg, comprising 0.080 kg SH, 0.060 kg CH, 0.00859 kg anhydrous SS, and 0.00750 kg dry NS. In this scenario, P8-6-2 required approximately 16.0% less transported admixture mass than P0.

In the experimental formulations, the combined mass of separately added water and water contained in the NS dispersion was 0.270 kg per kilogram of dry simulated lunar soil. The commercial NS dispersion contained 15 wt.% solids; therefore, 0.00750 kg of dry NS corresponded to 0.0500 kg of commercial dispersion. The water contained in this dispersion replaced an equivalent amount of separately added mixing water. The calculations in this section therefore assume that lunar regolith and process water are obtained on the lunar surface, whereas SH, CH, anhydrous SS, and dry NS are transported from Earth.

For the economic calculations, the procurement prices of SH and CH were approximately 1.5 USD/kg and 2 USD/kg, respectively. The price of anhydrous SS was approximately 2.5 USD/kg. The commercial NS dispersion price was approximately 70 USD/kg, equivalent to a dry-solids procurement price of 466.67 USD/kg based on its 15 wt.% solids content. Using these prices, the raw-material costs of P0 and P8-6-2 were 0.3344 USD and 3.7615 USD, respectively. At the reference Earth–Moon transportation price of 75,000 USD/kg [8], the corresponding transportation costs were 13,932.69 USD and 11,706.73 USD. Including raw-material costs, the combined costs were 13,933.02 USD and 11,710.49 USD, respectively. Thus, under this calculation scenario, P8-6-2 achieved a saving of approximately 2222.53 USD relative to P0 for each kilogram of simulated lunar soil solidified. The results are presented in Table 8.

To examine how declining Earth–Moon transportation costs affect the economic comparison, the transportation price at which the two formulations have equal combined costs was calculated while holding the above raw-material procurement prices constant. Compared with P0, P8-6-2 incurred approximately 3.4271 USD in additional raw-material costs while reducing the transported mass by approximately 0.02968 kg. Consequently, the combined raw-material and transportation costs of the two formulations become equal when the transportation price falls to approximately 115.47 USD/kg. Within the specified calculation scenario, P8-6-2 has a cost advantage above this price; below it, the transportation-cost saving is insufficient to offset the additional raw-material cost. This indicates that, at fixed raw-material prices, the cost advantage of P8-6-2 can persist following a substantial reduction in Earth–Moon transportation costs, although it remains dependent on the actual transportation price.

To incorporate mechanical performance into the economic comparison, a compressive-strength-normalized cost index was calculated:

(2) $$I_{C}=\frac{C}{f_{C}}$$

where *C* is the combined raw-material and transportation cost per kilogram of dry simulated lunar soil, and *f_c_* is the measured 28-day compressive strength. The index is expressed in USD/(kg soil·MPa) and compares cost relative to compressive performance on a fixed soil-mass basis. At the reference transportation price of 75,000 USD/kg, the compressive-strength-normalized costs of P0 and P8-6-2 were 485.47 and 323.94, respectively, corresponding to a reduction of approximately 33.3%. This analysis uses the existing measured strengths as performance benchmarks for the anhydrous supply scenario and assumes that comparable performance can be obtained using anhydrous SS and redispersed dry NS when the effective chemical inputs and total water content are matched. The actual performance associated with this supply arrangement requires further verification.

The lunar-surface water-supply scenario is primarily based on the potential for process-water recycling and the prospective utilization of water-ice resources in the lunar polar regions [71]. Our previous study showed that the water recovery rate of SLSG exceeded 96% after 3-day of curing and increased to 98.5% after 7-day [23]. Wang et al. [72] similarly reported that most of the water used in SLSG preparation could be recovered. These findings provide a basis for developing enclosed preparation and curing systems in which released water vapor is collected, condensed, treated, and reused. In the supply scenario adopted in this section, initial water and subsequent make-up water are assumed to be obtained from accessible lunar resources, while process-water recycling reduces the demand for continued water supply.

Implementing this scenario would still require energy for water extraction and purification, water transport and pumping, and water-vapor collection, condensation, and recycling. Condensation also requires suitable heat-rejection conditions, while the energy demand of in situ water extraction depends on the location, water content, and accessibility of the resource. Therefore, a high water recovery rate does not directly imply low system energy consumption. These energy requirements and the associated equipment inputs have not been included in the raw-material and transportation cost comparison presented in this section. Future studies should combine repeated water-recycling experiments with assessments of water quality, make-up water demand, NS redispersion, and material performance, followed by further analysis of process energy consumption and equipment requirements. The results presented here therefore reflect potential transportation-cost and resource-efficiency benefits under the anhydrous-admixture transportation and lunar-surface water-supply scenario.

### 4.2. Differential Analysis of Activation Mechanisms

The combined macro- and microscale characterization results provided a basis for interpreting the distinct roles of SH, CH, and SS in the activation of simulated lunar soil.

SH appeared to play a major role in precursor depolymerization. The ICP-AES results showed that the SH dosage had the greatest influence on the measured Al and Si concentrations: when the SH dosage was reduced from 10 wt.% to 6 wt.%, the measured Al and Si concentrations decreased by 79.1% and 93.9%, respectively. This trend may be associated with the high concentration of OH^−^ provided by SH, which facilitates the disruption of Si-O-Si/Al bonds. XRD further showed that the diffraction-peak intensities of phases such as quartz decreased at higher SH dosages, supporting the proposed role of SH in the reaction of the simulated lunar soil. Consequently, increasing the SH dosage was associated with higher compressive and flexural strengths of NS-SLSG, while the range analysis identified SH as one of the factors with a comparatively large influence on mechanical properties.

CH primarily promotes polycondensation through the sustained release of Ca^2+^ during the geopolymerization process. The introduced Ca^2+^ possesses a stronger charge neutralization capacity than Na^+^, enabling electrostatic attraction of aluminate monomers and promoting their condensation with silicate monomers via a nucleation effect to form C-A-S-H gel. FT-IR and TG-DSC analyses revealed that the CH-rich groups exhibited higher Si–O–T absorption peak intensities and increased chemically bound water content, indicating a higher degree of geopolymerization. EDS, MIP, and SEM analyses further demonstrated that increasing CH dosage significantly elevated the Ca/Si ratio, promoted the formation of C-A-S-H gel, and reduced porosity, thereby driving microstructural densification. Therefore, CH likewise constitutes one of the dominant factors governing mechanical properties.

Within the formulation range investigated in this study, SS exhibited a comparatively limited regulatory effect on the mechanical properties of NS-SLSG. Because all nine orthogonal mixtures contained 0.75 wt.% NS, the additional reactive silica supplied by NS may have brought the available silica content of the system closer to an appropriate level, thereby reducing the marginal response of compressive strength to variations in SS dosage. However, our previous study on TJ-1-based SLSG without NS similarly found that the relative influence of SS on mechanical properties was smaller than those of SH and CH [73], indicating that the limited influence of SS observed in the present study cannot be attributed entirely to the additional reactive silica introduced by NS. This may also be related to the intrinsic Si/Al ratio of approximately 2.9 in TJ-1, which provides a relatively favorable silica compositional basis for gel-network formation, as well as to the distinct roles of the different activator components. In comparison, SS primarily serves as a supplementary silica source and contributes to the regulation of slurry workability. As the available silica in the system gradually approaches an appropriate level, the marginal strength gain resulting from further increases in SS dosage may correspondingly diminish.

In summary, the geopolymerization of NS-SLSG is governed by a synergistic balance between SH-driven depolymerization and CH-promoted polycondensation, with SS playing an auxiliary role in workability regulation and supplementary silicon supply. Among the investigated formulations, P8-6-2 provided a comparatively favorable balance among these effects and exhibited the most favorable combination of reaction-product development, pore structure, and mechanical performance.

Although the combined macro- and microscale analyses provide complementary evidence for the proposed activation mechanism, certain limitations remain. ICP-AES measurements are useful for comparing the relative elemental release under different activator conditions. However, because the suspensions were allowed to equilibrate for three days before analysis, the dissolved elements may have simultaneously undergone precipitation or secondary-phase formation, and the measured elemental concentrations may therefore reflect multiple concurrent processes rather than dissolution alone. Regarding microstructural characterization, FT-IR and XRD are qualitative techniques that provide information on functional groups and crystalline phases but cannot precisely quantify the degree of polymerization. Although TG-DSC-DTG may reflect the relative gel content, the measured mass loss cannot be unambiguously assigned to a specific gel type. The elemental ratios obtained by EDS can be used to infer possible gel types, but the results are affected by the selection of analysis points and may not fully represent the overall gel composition. Therefore, the mechanistic conclusions of this study should be regarded as reasonable interpretations based on the available evidence rather than definitive conclusions.

In addition, the specimens were cured at 100 °C and tested under terrestrial laboratory conditions, without considering lunar vacuum, thermal cycling, radiation, and other relevant environmental factors. The preliminary economic and resource-efficiency comparison was mainly based on admixture mass, raw-material prices, and Earth–Moon transportation costs, without including curing energy, water demand, NS production, and its associated inputs. Future studies will incorporate more representative lunar environmental exposures, higher-resolution and quantitative microstructural characterization, structural-scale testing, and life-cycle assessment to further evaluate the long-term performance and resource efficiency of NS-SLSG.

## 5. Conclusions

This study optimized the composite alkali-activator ratio for NS-SLSG preparation, with particular attention to the measured Si, Al, and Ca concentrations under different activation conditions. Flow, mechanical properties, and mass–strength efficiency of NS-SLSG were evaluated. Microstructural characterization indicated differences associated with SH, CH, and SS activation and highlighted microstructural variations in NS-SLSG across the investigated ratios. Key findings include:

(1) Under alkali activation, the apparent elemental-release response generally followed the order SH > SS > CH. The SH dosage had a comparatively strong influence on the measured Al and Si concentrations, and reducing the SH dosage was accompanied by marked decreases in both values. The SS dosage influenced the measured Si concentration, while a CH dosage of ≥4 wt.% was associated with comparatively stable measured Ca concentrations within the tested range.
(2) Reducing composite alkali-activator dosage deteriorates flow properties, primarily regulated by SH and SS in the order SH > SS > CH. Optimal flow performance of 152.5 mm is achieved at a total activator dosage ≤ 16 wt.%, and NS-SLSG was prepared with 8 wt.% SH, 2 wt.% CH, 6 wt.% SS, and 0.75 wt.% NS.
(3) The mechanical properties of NS-SLSG deteriorated with reduced composite alkali-activator dosages and were primarily influenced by SH and CH. Compressive strength influence follows CH > SH > SS, while flexural strength follows SH > CH > SS. At a total activator dosage of ≤16 wt.%, NS-SLSG containing 8 wt.% SH, 6 wt.% CH, 2 wt.% SS, and 0.75 wt.% NS exhibited the best mechanical performance among the investigated formulations, with 28-day compressive and flexural strengths of 36.15 and 8.26 MPa, respectively, showing potential for future load-bearing applications in lunar research station construction.
(4) At a total composite alkali-activator dosage of ≤16 wt.%, incorporating NS improves the flow properties of SLSG. The high reactivity of NS effectively compensates for the insufficient activation capacity under low-alkali activator dosage; with the addition of 0.75 wt.% NS, a 23.9% reduction in total admixture mass in the experimental formulation was achieved while maintaining enhancements of 26.0% in compressive strength and 19.5% in flexural strength. This approach improves mass–strength efficiency, showing potential economic and resource-efficiency benefits for lunar construction.
(5) Changes in the composite alkali-activator ratio did not significantly affect the chemical bond composition or physical phase composition of NS-SLSG, but generated local variations in gel elemental composition and distinct pore structures. The combined characterization results suggest that SH may promote the availability of Si- and Al-containing species under highly alkaline conditions, while its Na^+^ contributes to Na-rich aluminosilicate reaction products. CH may facilitate the formation of Ca-containing reaction products, whereas SS mainly influences Si supply and workability. Increasing SH and CH dosages was associated with variations in reaction-product composition and reductions in porosity and coarse-pore content.

## Figures and Tables

**Table 1:** ICP-AES test group.

Sample	SH (wt.%)	CH (wt.%)	SS (wt.%)
P6-0-0	6	0	0
P0-6-0	0	6	0
P0-0-6	0	0	6
P10-6-6	10	6	6
P10-6-4	10	6	4
P10-6-2	10	6	2
P10-4-6	10	4	6
P10-2-6	10	2	6
P8-6-6	8	6	6
P6-6-6	6	6	6

Note: The mass percentage is that of the simulated lunar soil (1100 g) with which the specimens were prepared.

**Table 2:** Orthogonal test group.

Sample	TJ-1 (g)	SH (wt.%)	CH (wt.%)	SS (wt.%)	NS (wt.%)
P0	1100	10	6	6	0
P10-6-6	1100	10	6	6	0.75
P10-4-2	1100	10	4	2	0.75
P10-2-4	1100	10	2	4	0.75
P8-6-2	1100	8	6	2	0.75
P8-4-4	1100	8	4	4	0.75
P8-2-6	1100	8	2	6	0.75
P6-6-4	1100	6	6	4	0.75
P6-4-6	1100	6	4	6	0.75
P6-2-2	1100	6	2	2	0.75
P1	1100	8	6	2	0

Note: X, Y, and Z in PX-Y-Z represent the mass percentages of SH, CH, and SS, respectively, and P8-6-2 means 8 wt.% SH, 6 wt.% CH, and 2 wt.% SS were blended in. All percentages are mass percentages with simulated lunar soil.

**Table 3:** *Ri* of flowability.

Sample	SH	CH	SS
*K*_1_	152.83	147.17	155.00
*K*_2_	147.00	137.83	132.33
*K*_3_	125.17	140.00	137.67
*R_i_*	27.66	9.34	22.67

**Table 4:** *R_i_* of 28-day compressive strength for NS-SLSG.

Sample	SH/	CH/	SS/
*K*_1_	29.59	31.91	23.20
*K*_2_	25.12	20.76	22.98
*K*_3_	13.78	15.82	22.31
*R_i_*	15.81	16.09	0.89

**Table 5:** *R_i_* of 28-day flexural strength for NS-SLSG.

Sample	SH/	CH/	SS/
*K*_1_	7.36	7.69	6.06
*K*_2_	6.10	5.01	5.42
*K*_3_	3.06	3.82	5.05
*R_i_*	4.30	3.87	1.01

**Table 6:** EDS elemental test results.

Sample	Na (%)	Al (%)	Si (%)	Ca (%)	Al/Si	Na/Al	Ca/Si
P10-4-2	5.16	8.14	23.63	5.64	0.34	0.63	0.24
P10-2-4	4.04	4.45	20.36	1.42	0.22	0.91	0.07
P8-6-2	6.78	9.71	21.78	6.74	0.45	0.70	0.31
P6-4-6	9.01	2.88	16.23	15.00	0.18	3.13	0.92

**Table 7:** Pore-structure characteristic parameters of NS-SLSG.

Sample	Porosity (%)	Average Pore Size (nm)	Optimal Pore Size (nm)	Median Pore Size (nm)
P10-4-2	39.29	252.02	198.73	135.09
P10-2-4	38.99	493.07	2273.08	178.54
P8-6-2	35.88	231.88	198.58	125.57
P6-4-6	39.82	494.91	9282.99	127.42

**Table 8:** Preliminary cost-benefit comparison based on raw-material and transportation costs.

Sample	Raw Materials	Transportation	Total Cost
P0	0.3344	13,932.69	13,933.02
P8-6-2	3.7615 (+3.4271)	11,706.73 (2225.96)	11,710.49 (2222.53)

Note: All quantities and costs are expressed per kilogram of dry simulated lunar soil. SS masses were converted to anhydrous equivalents using the nominal chemical formula. Locally supplied process water includes the 0.270 kg water input and the additional water required to replace the crystallization water originally supplied by SS; this water is excluded from Earth–Moon transportation mass. The NS procurement price is expressed on a dry-solids-equivalent basis. Calculations used unrounded values.

## Data Availability

The original contributions presented in this study are included in the article. Further inquiries can be directed to the corresponding author.

## Abbreviations

The following abbreviations are used in this manuscript:

SLSG	Simulated lunar soil-based geopolymer
NS	Nano-silica
SH	Sodium hydroxide
CH	Calcium hydroxide
SS	Sodium silicate
